# Protein Activity Sensing in Bacteria in Regulating Metabolism and Motility

**DOI:** 10.3389/fmicb.2019.03055

**Published:** 2020-01-17

**Authors:** Alejandra Alvarado, Wiebke Behrens, Christine Josenhans

**Affiliations:** ^1^Max von Pettenkofer-Institute, Ludwig Maximilian University of Munich, Munich, Germany; ^2^German Center for Infection Research (DZIF) Partner Site Munich, Munich, Germany; ^3^Institute of Medical Microbiology and Hospital Epidemiology, Hannover Medical School, Hanover, Germany

**Keywords:** flux sensing, energy taxis, protein-protein interaction, iron-sulfur cluster, TCS, metabolism, flagellar motility, protein conformation

## Abstract

Bacteria have evolved complex sensing and signaling systems to react to their changing environments, most of which are present in all domains of life. Canonical bacterial sensing and signaling modules, such as membrane-bound ligand-binding receptors and kinases, are very well described. However, there are distinct sensing mechanisms in bacteria that are less studied. For instance, the sensing of internal or external cues can also be mediated by changes in protein conformation, which can either be implicated in enzymatic reactions, transport channel formation or other important cellular functions. These activities can then feed into pathways of characterized kinases, which translocate the information to the DNA or other response units. This type of bacterial sensory activity has previously been termed *protein activity sensing*. In this review, we highlight the recent findings about this non-canonical sensory mechanism, as well as its involvement in metabolic functions and bacterial motility. Additionally, we explore some of the specific proteins and protein-protein interactions that mediate protein activity sensing and their downstream effects. The complex sensory activities covered in this review are important for bacterial navigation and gene regulation in their dynamic environment, be it host-associated, in microbial communities or free-living.

## Canonical and Non-Canonical Sensory Systems in Bacteria

Bacteria face constant challenges in their changing environments. Nonetheless, bacteria can adapt and ensure their survival by employing several strategies. They can modify their cellular activities and, for instance, suppress the synthesis of certain proteins, or in turn they can produce enzymes that are required to metabolize available nutrients. Additionally, some bacteria can transition into more resistant phases (i.e., spores) in the absence of appropriate nutrition (Armitage, 1997). Another important strategy is flagella-driven motility, entailing the sensing of environmental cues and displacement of the cell to more favorable environments (Porter et al., 2011). These adaptation means are arguably analogous to the “flight or fight” responses described in higher animals (Lee and Wang, 2019; Russell and Lightman, 2019).

Vast repertoires of sensory systems that mediate these diverse and temporally distinct responses have evolved in bacteria. Well-characterized kinase-dependent systems important for sensing and signaling are two-component systems (TCSs), consisting of a sensor kinase or multi-kinase networks (Francis and Porter, 2019) and a corresponding response regulator (Capra and Laub, 2012; Groisman, 2016; Zschiedrich et al., 2016; Willett and Crosson, 2017). TCSs allow bacteria to sense a wide range of stimuli and induce a multitude of downstream effects, ultimately affecting gene expression (Gao and Stock, 2009; Desai and Kenney, 2017). Usually, bacteria encode a variety of different sensor kinases and TCSs, and the diversity and number of kinases can vary enormously between species, as they match the different needs within bacteria habitats (Ashby, 2004; Krell et al., 2010; Porter et al., 2011; Francis and Porter, 2019).

**FIGURE 1:**
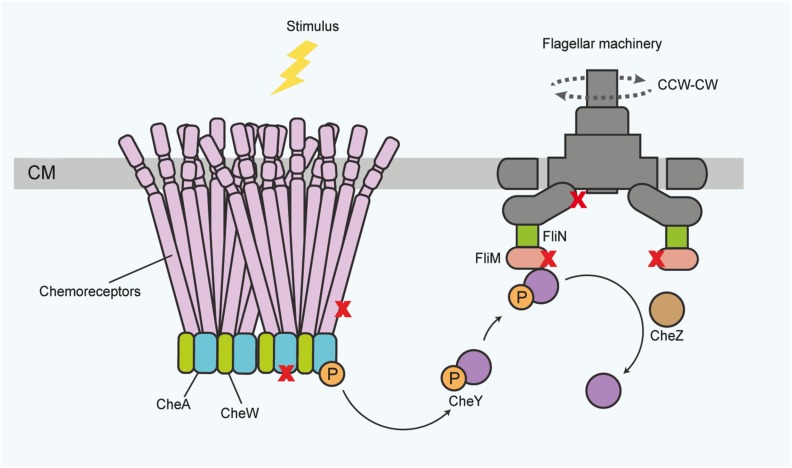
General scheme of the canonical chemotactic sensing and signaling pathway in the bacterial membrane for governing motility functions, which are also accessible for protein activity sensing. Receptor proteins associate in large complexes of trimers of dimers, spanning the inner membrane toward the cytoplasm. In the cytoplasm, the receptors associate with the histidine kinase CheA and the adaptor protein CheW. Upon stimulus binding, CheA autophosphorylates. Subsequently the response regulator protein CheY activates and translocates the information to the flagella motor proteins, ultimately inducing a change in the direction of flagella rotation (dotted lines) or flagellar speed. The chemotaxis components, and flagellar basal body proteins, for instance the FlhA and FliM proteins, also provide a regulatory platform for diverse activity inputs and protein-protein interactions (symbolized by X). Phosphorylated CheY can be dephosphorylated for signal termination by various proteins, for instance CheZ. CM, cytoplasmic membrane; P, phosphoryl group. CW – clockwise; CCW – counterclockwise (for the direction of flagellar rotation). The top of the panel points to the outside of the bacterial envelope.

Canonical TCSs comprise one or several regulatory kinases and a cognate response regulator, which is phosphorylated upon sensor activation (Bourret and Silversmith, 2010; Groisman, 2016; Desai and Kenney, 2017). Canonical sensors can be membrane-bound as valid for most sensor kinases (Bourret and Silversmith, 2010; Desai and Kenney, 2017; May et al., 2019), periplasmic (Tschauner et al., 2014; Wiech et al., 2015; Matson et al., 2017; Masilamani et al., 2018), or cytoplasmic (Galperin, 2018; Osman et al., 2019). A widely studied specific example of TCS is the bacterial chemotactic sensory module, consisting of a transmembrane sensing unit, the histidine kinase CheA and the basic signaling protein CheY (Kirby, 2009; Porter et al., 2011; Parkinson et al., 2015). This system governs bacterial motility by modulating flagellar rotation and swimming direction over time, triggered by input from specialized taxis sensors (Figure 1).

We would also like to mention quorum sensing as one important subsystem of bacterial sensory activity. Quorum sensing is mediated by autoinducer (AI) molecules in concert with specific receptors, which allow bacteria to communicate in a population [summarized in two recent reviews (Papenfort and Bassler, 2016; Whiteley et al., 2017)]. The first major family of AI receptors comprises the transmembrane dimeric receptors which phosphorylate a DNA-binding response regulator, similar to canonical TCS. AI receptors of a second major family are cytoplasmic (LuxR-type family) and bear similarity to DNA-binding response regulators (Papenfort and Bassler, 2016). All quorum sensing modules seem to directly link exclusively to transcriptional regulation.

In addition to these well-characterized sensory systems and families, an increasing number of reports highlight the importance of *protein activity sensing* (Mascher, 2014). These non-canonical sensory processes can link to canonical sensory modules and their importance and means of action are the focus of this review.

Non-canonical sensory processes involve protein conformational changes and, frequently, specific protein-protein interactions, which affect the downstream signaling relays and can also feed into the known kinase pathways. Furthermore, several accessory proteins of important functions in non-canonical sensing have been uncovered, including examples of proteins whose activity is sensed as an input signal of a signaling cascade. In the next paragraphs we focus primarily on recent examples which illustrate that changes in protein functionality and conformation at any given moment can be of specific importance for non-canonical sensing processes, which particularly regulate metabolism and motility, and in some cases, even act entirely independently of a TCS.

## Regulatory Influence of Membrane Transport Proteins and Intracellular Protein Activities in Metabolic Homeostasis and Antibacterial Resistance

The concept of co-opting accessory proteins such as transport proteins for sensory purposes in the downstream activation of TCSs has been described in several bacterial species, primarily *Escherichia coli* and *Bacillus subtilis* (see below). These co-regulatory processes frequently involve the maintenance of metabolic homeostasis. Mostly, they occur at the bacterial membrane, involving transport proteins of metabolically important substrates and their regulation, thus playing a role in the co-regulation of metabolic functions by membrane-bound TCSs. Some protein activities, possibly linked to freely diffusing metabolic substrates, also contribute to cytoplasmic sensing as highlighted below.

**FIGURE 2:**
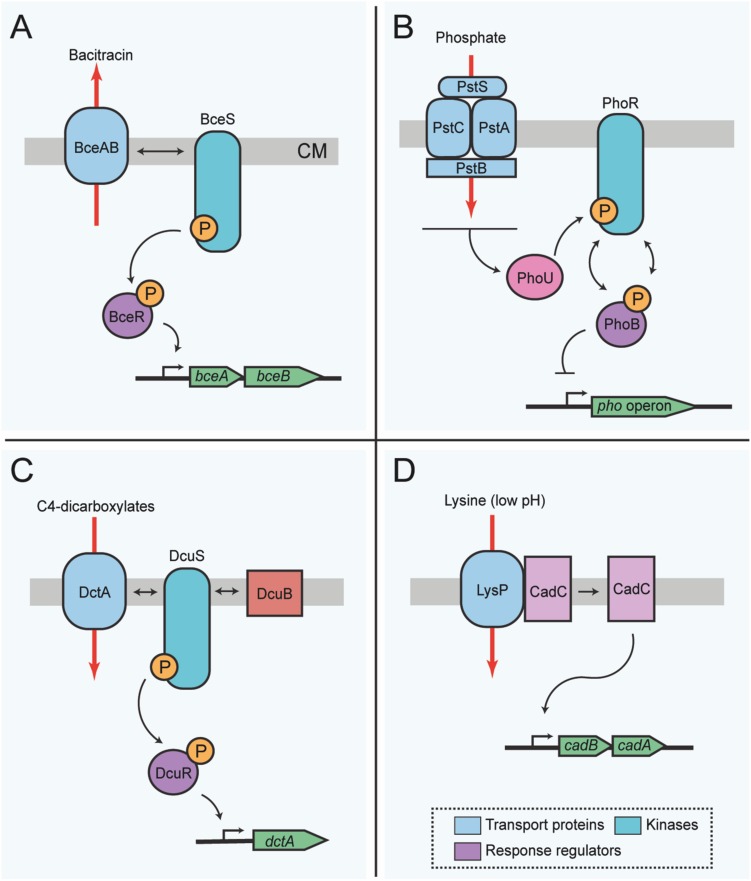
Membrane transport functions involved in bacterial protein activity or flux sensing for metabolic homeostasis. **(A)** Flux-sensing by the BceRS-BceAB system described in *B. subtilis* for detoxification of bacitracin. The transporter proteins BceAB sense bacitracin, and such activity influences activation of the kinase BceS, which in turn activates the response regulator protein BceR. Active BceR recruits RNA polymerase for the transcription of genes required for the synthesis of more transporter proteins needed for detoxification of the cell. Thus, changes in the activity of the transporters is signaled from BceS to BceR, and the amount of BceR-P is proportional to the bacitracin load on the transporters. In this way, the transporter proteins are both, the sensor and the means for antibiotic resistance. **(B)** Sensing of external phosphate in *E. coli* via PhoR-PhoB. The phosphate transporter complex PstCAB perceives external phosphate levels. Phosphate binds to the transporter complex, which then transfers this information to the histidine kinase PhoR via the chaperone protein PhoU. In its default state, PhoR autophosphorylates and subsequently transfers the phosphoryl group to the response regulator protein, PhoB. However, upon phosphate binding to PstS, phosphorylation of PhoR and thus PhoB is inhibited, resulting in a concomitant decrease of the transcription of the *pho* regulon. **(C)** Sensor switch of DcuS-DcuR or DctS-DctR in *E. coli* and *B. subtilis*, respectively. Sensing of C4-dicarboxylate compounds occurs indirectly through the kinase DcuS, which complexes with transport proteins, in this example DctA and the accessory protein DcuB. Complexing of the transport protein and DcuS activates kinase activity, initiating the phoshorelay to the response regulator protein, DcuR, and ultimately inducing expression of genes for the synthesis of more transporter proteins that will eventually complex with the kinase for an increased uptake of C4-dicarboxylates. Several genes important for the synthesis of proteins required to degrade external C4-dicarboxylates are under the control of this switch; however, for simplicity only *dcuB* is shown. **(D)** Regulatory interplay between LysP and CadC. In the presence of lysine (K) at low pH (≤5.8) the lysine permease LysP interaction with the sensor protein CadC is prevented, allowing the release of CadC, which in turn induces the expression of *cadAB*. Red arrows indicate the direction of transport. Box includes the color conventions of shared elements between systems. CM, cytoplasmic membrane; P, phosphoryl group. The top of each panel points to the outer face of the bacterial envelope.

Most classes of antibacterial compounds act on conserved central metabolic functions which directly or indirectly threaten bacterial survival and proliferation. Therefore, the maintenance of metabolic homeostasis and antimicrobial resistance mechanisms are tightly interconnected. A novel mode of protein activity sensing, termed *flux sensing*, involves protein conformational changes upstream of a TCS cascade (Fritz et al., 2015). In the study that first described and named this mechanism, the authors determined that the cue sensed by the bacteria is the presence of the antibiotic bacitracin. Bacitracin acts on the bacterial cell wall, and thereby indirectly impairs physiological functions of the bacterial cell (Clemens et al., 2018). The presence of bacitracin is not sensed directly by a receptor, but indirectly, via the activity of the ABC transporter BceAB of *B. subtilis*, which is located in the bacterial membrane and mediates antibiotic resistance by driving the efflux of bacitracin (Bernard et al., 2007; Dintner et al., 2014; Fritz et al., 2015). Upon activation of the transporter by its substrate, BceAB directly interacts with the histidine kinase BceS of the TCS BceS/BceR. The external and cytoplasmic levels of the antibiotic bacitracin influence the activity of the ABC transporter proteins BceAB and as a direct consequence, regulate transcription of its own genes via the downstream signaling mediated by the TCS BceS/BceR (Figure 2A) (Dintner et al., 2011; Fritz et al., 2015). After the sensing process is initiated and upon activation by the kinase, the response regulator BceR binds DNA in order to increase the transcription of the transporter genes and other related functions (Fritz et al., 2015; Radeck et al., 2017).

Besides *B. subtilis*, similar complex activity sensing systems to detect the presence of antibiotics or antimicrobial peptides, exist in other Gram-positive *Firmicutes* (Ouyang et al., 2010; Coumes-Florens et al., 2011; Dintner et al., 2011; Muzamal et al., 2014). In fact, the impact of ABC transporters on TCS sensing may be widely distributed, in particular in the *Firmicutes*, where dozens of genes encoding for ABC transporters have been found in adjacent regions to, or even within, TCS operons (Mascher et al., 2006; Tetsch and Jung, 2009; Dintner et al., 2011; Gebhard and Mascher, 2011; Gebhard, 2012).

Another example of transport-dependent metabolic regulation in Gram-negative bacteria is the *E. coli* ABC phosphate transporter PstSCAB, which indirectly co-regulates the activity of the histidine kinase PhoR, via the linker protein PhoU (Gardner et al., 2014; Västermark and Saier, 2014), which does not itself contribute to the transporter function. Experimental evidence suggests that physical interaction between the transporter subunit PstB, along with PhoR and the auxiliary protein PhoU, ensures that phosphate limitation or phosphate repletion conditions are correctly relayed to the response regulator PhoB (Gardner et al., 2014; Vuppada et al., 2018). PhoR does not have a canonical periplasmic sensor domain. Instead PhoR is activated by the transporter complex PstSCAB via PhoU, and subsequently, activated PhoR relays information to the response regulator PhoB. In its phosphorylated state, PhoB activates gene expression of the *pho* regulon, including periplasmic alkaline phosphatase (Figure 2B) (Hsieh and Wanner, 2010). The default state of PhoB alone is a phosphorylated state, which can be modeled by deleting the genes encoding for the transporter proteins or *phoU*. Phosphate repletion in the presence of the ABC transporter then signals to induce phosphatase activation of the sensor kinase and dephosphorylation of PhoB (Wanner and Wilmes-Riesenberg, 1992).

Many bacteria preferentially utilize C4-carbohydrates in addition to hexoses for their metabolism. Among those, *E. coli* and *B. subtilis* C4 transporters are some of the best-characterized for auxiliary functions in signal transduction. In particular, the C4-dicarboxylate/orotate symporter DctA, which is a polarly localized non-ABC C4-carbohydrate transporter in *E. coli* (Scheu et al., 2014; Unden et al., 2016) and similarly in *B. subtilis* (Groeneveld et al., 2010; Graf et al., 2014), can form a sensory unit with a transmembrane sensor kinase. In *E. coli* this is the protein DcuS of the DcuS/DcuR TCS (Monzel et al., 2013). Upon DcuS activation by DctA, the response regulator is activated, leading to the upregulation of the DctA transporter (Figure 2C). Activity of the transporter itself seems not to be required for the sensing process, but DctA presence alone can provide an activity switch for the sensor kinase (Steinmetz et al., 2014). Kinase activity is dependent on the absolute protein levels of the transporter proteins, substrate availability and substrate binding to the transporter (Wörner et al., 2018), which are in turn sufficient to lead to a DctA conformational change that is possibly the signal transmitted to the kinase.

A similar mechanism with a close association of signal transfer and signal conversion by a transporter has also been described for *E. coli* DcuB, an aerobically active fumarate/succinate C4 antiporter, which also directly interacts with and activates the histidine kinase DcuS (Pappalardo et al., 2003; Kneuper et al., 2005; Kleefeld et al., 2009; Witan et al., 2012; Strecker et al., 2018; Wörner et al., 2018). Under aerobic conditions, DcuB can replace anaerobically active DctA as co-sensor for the TCS, such that under both divergent conditions, appropriate target genes can be activated by the response regulator. Interestingly, when both *dctA* and *dcuB* are absent, the kinase DcuS is completely deregulated and in a constitutively active state, even if no C4 transport substrates are present (Kleefeld et al., 2009).

In *B. subtilis*, the transporter DctA, an orthologous C4 transporter protein as the one reported in *E. coli*, activates the *B. subtilis* DctS/DctR TCS. In this case, a second accessory protein, DctB, which is a membrane co-receptor for C4-carbohydrates, has to be present as well to initiate the sensing process, probably acting as a tripartite sensory unit together with DctS and DctA, each binding at different domains of the sensory kinase (Graf et al., 2014).

Such co-regulatory mechanisms may not only be widespread for carbohydrate transporters, but also for amino acid transport proteins such as CadC, which is part of an *E. coli* one-component system that receives information on lysine availability from LysP, a lysine-specific membrane-associated amino acid permease (Figure 2D) (Tetsch and Jung, 2009; Haneburger et al., 2011; Rauschmeier et al., 2014; Brameyer et al., 2019). When lysine is available and the cell is under stress due to low pH, the sensor protein CadC activates the expression of the *cadBA* genes. CadA is a lysine decarboxylase, while CadB acts as a lysine/cadaverine antiporter (Fritz et al., 2009). Thus, lysine is converted to cadaverine via CadA-mediated decarboxylation reaction, which ultimately raises the intracellular pH. Meanwhile, CadB transports lysine into the cell and cadaverine out. The presence of lysine is transduced to CadC via LysP (Tetsch et al., 2008; Rauschmeier et al., 2014). LysP inhibits the dimerization of CadC under non-inducing conditions, but when the external pH drops, CadC undergoes structural modifications that permit its binding and consequent activation of the *cadBA* operon (Haneburger et al., 2011, 2012; Lindner and White, 2014; Buchner et al., 2015).

One example of protein activity sensing concerning the membrane but not immediately involved in transport evolved with the nitrate sensor NtrB in *E. coli* (Buelow and Raivio, 2010). The NtrB activating system is indirectly associated with ammonium transport and has recently been termed a “Level and Activity Proportional Integral” (LA-PI) sensor-controller, optimized for performance under stepwise changing environmental conditions (Mairet, 2018). Specifically, the LA-PI pattern of feedback control refers to a specific feedback system which acts by both, transcriptional and post-translational, feedback regulation toward the same regulatory protein (any given biomolecular control protein), which thereby acts as a proportional integral (PI) controller (Mairet, 2018). Modeling of the feedback response of active sensory proteins to their substrates demonstrated that indeed feedback control plays an important role in the response by various sensors (Mairet, 2018). Chemotactic responses have been modeled as direct zero integral feedback control (Dufour et al., 2014).

**FIGURE 3:**
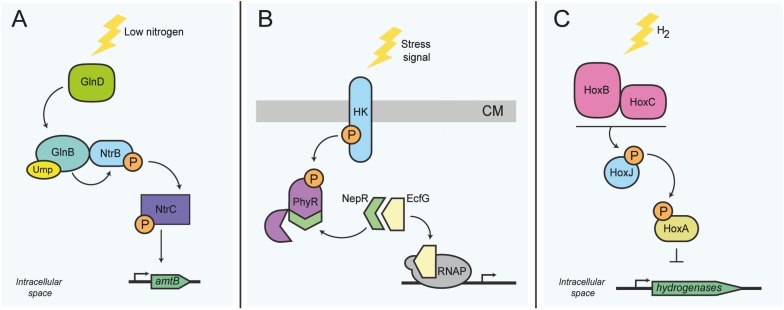
Activity sensing in intracellular bacterial functions related to metabolism. **(A)** Nitrogen stress response in *E. coli*. At low nitrogen levels, GlnD uridylates GlnB. GlnB-UMP subsequently activates the TCS NtrB/NtrC. The intracellular kinase NtrB autophosphorylates, and in turn phosphorylates the DNA-binding transcriptional regulator NtrC; activation of NtrC is needed for the transcription of several genes, including *amtB*. **(B)** Transcription control switch of the general stress response in alphaproteobacteria. Stress sensing activates autophosphorylation of the histidine kinase, which in turn phosphorylates the regulator protein PhyR. Phosphorylation of PhyR induces its conformational change, allowing the sequestration of the anti-sigma factor NepR, consecutively releasing the transcriptional regulator sigma-EcfG, which permits the transcription of general stress response genes. **(C)** H_2_ sensing in *R. eutropha*. Expression of genes required for the synthesis of hydrogenases is controlled via sensing of molecular hydrogen by the intracellular Ni^2+^Fe^2+^ hydrogenases HoxB and HoxC, which then interact presumably with the histidine kinase HoxJ. HoxJ, when activated by HoxBC, prevents HoxA-mediated transcription of several hydrogenase genes (horizontal line). HK, histidine kinase; RNAP, RNA polymerase; CM, cytoplasmic membrane; P, phosphoryl group; UMP, Uridine monophosphate group. The top of panel **B** points to the outer face of the bacterial envelope.

The intracellular kinase NtrB is connected physically to the regulatory PII protein GlnB and controls, among others, the biogenesis and activity of the AmtB ammonium importer (Huergo et al., 2013; Mairet, 2018). The bifunctional glutamine-sensing protein GlnD uridylates GlnB and thereby regulates GlnB conformation and direct GlnB-NtrB binding (Zhang et al., 2010). During states of high intracellular ammonium concentration, GlnB in complex with NtrB represses transporter activity and transcriptional activation of downstream genes via the response regulator NtrC (Figure 3A) (Zimmer et al., 2000; Huergo et al., 2013). In order to initiate the transcription of genes required for nitrogen assimilation, the phosphorylated form of NtrC binds DNA. Ultimately this interaction facilitates access of the RNA polymerase subunits to the promoter region (Hervas et al., 2009). Similar signaling pathways also exist in other bacteria, such as *Sinorhizobium meliloti*, *Rhodospirillum rubrum*, and *Azospirillum brasilense* (Zhang et al., 2010; Inaba et al., 2015; Hauf et al., 2016).

More complex activity sensing can also involve anti-sigma factors, which generally require conformational change to associate or disassociate with their cognate binding proteins. One common sensory module involves the interaction between the response regulator PhyR and the anti-sigma factor NepR of *Caulobacter crescentus*. Kinase activation upon stress sensing permits phosphorylation of PhyR. Activation of PhyR induces its conformational change that permits binding of the anti-sigma factor NepR; this in turn allows the release of the sigma factor EcfG and the consequent transcription of general stress response genes (Figure 3B) (Herrou et al., 2010; Fiebig et al., 2015). This system is widespread amongst alphaproteobacteria and controls the general stress response (Fiebig et al., 2015).

In order to illustrate that protein activity sensing can also be crucial for exclusively intracellular sensory capacities, we highlight the hydrogenase HoxBC of *Ralstonia eutropha*. The HoxBC complex converts hydrogen into protons and electrons and acts as a cytoplasmic hydrogen sensor in connection with the histidine kinase HoxJ and the regulator protein HoxA. HoxA acts as the main DNA-binding transcriptional activator of hydrogenase genes found within several operons present in *R. eutropha* (Figure 3C) (Buhrke et al., 2004; Jugder et al., 2015). The exact sensory mechanism is still under investigation, but likely involves enzyme activity changes under conditions of oxidative stress. Oxidation can cause structural alterations to the iron-sulfur clusters present in HoxBC (Löscher et al., 2010), which is likely to lead to protein conformational changes or unfolding, which might then indirectly mediate hydrogen sensing. The differential interaction of the HoxBC complex with the kinase HoxJ is supposed to play a role in this sensing mechanism (Buhrke et al., 2004). This alternative display of two divergent functions is not unique of these proteins, as it has been shown for other proteins such as the cytoplasmic enzyme aconitase in *E. coli*, *Helicobacter pylori* and other organisms (Kiley and Beinert, 2003), which will be discussed in detail further ahead.

Altogether, these examples not only point at the major role of non-canonical sensory proteins in the activation of one and TCSs, they also highlight the relevance of activity sensing in the regulatory responses to diverse extra- and intracellular stimuli. Transport proteins as well as intracellular sensors not only mediate the activation of response regulator units, their activity also coordinates transcriptional regulation of downstream components that ultimately control the cellular response to antibiotics or metabolic maintenance. We speculate that the possibility to elicit a response via protein activity sensing could attend to the need for subtle but rapid changes in the face of continuous environmental challenges. This could have led to an evolutionary selection of protein activity sensing, which seems to convey a larger flexibility to the regulatory modules.

## Protein-Protein Interaction Sensing in Flagellar Assembly Regulation

The expression of flagellar genes in various bacteria is organized in a complex regulatory hierarchy. Although the number and organization of flagellar genes in the genome varies between bacterial genera, some analogies in hierarchical structure and feedback mechanisms are maintained (Chevance and Hughes, 2017). This hierarchical structure can include the (σ80) housekeeping sigma factor as well as the subordinate regulators σ54 and σ28 for middle and late flagellar genes, and further regulators, such as the anti-σ28 factor FlgM. FlgM provides the last assembly feedback step, which releases the late sigma factor FliA to produce late flagellar structural proteins such as the flagellins if the flagellar basal body and hook are completed (Chevance and Hughes, 2017). For instance, in the Epsilon-proteobacterium *H. pylori*, which has a three-tiered regulatory hierarchy of flagellar expression and assembly, including three sigma factors and a FlgM feedback loop (Niehus et al., 2004), researchers have found that several flagellar structural proteins are involved in flagellar regulation. These proteins include the basal body component FlhA, an essential part of the basal body and the assembly platform (Schmitz et al., 1997; Rust et al., 2009; Minamino et al., 2016a, b; Terahara et al., 2018) as well as FlhF, considered a signal recognition particle (SRP)-like protein important for flagellar localization (Bange et al., 2007; Kazmierczak and Hendrixson, 2013; Kondo et al., 2017); additionally the TCSs FlgS-FlgR and ArsS-ArsR are involved (Pflock et al., 2006; Loh et al., 2010; Marcus et al., 2016; Xiong et al., 2019).

These components, together with their known transcription factors, contribute to an expression cascade, which allows the coordinated buildup of the complex flagellar structure (Figure 1), involving early (class 1), intermediate (class 2 and 3), and late (class 3) flagellar genes (Niehus et al., 2004; Lertsethtakarn et al., 2011). It is well established in various species that a deficiency of *flhA* by directed mutagenesis leads to the downregulation of numerous middle and late flagellar transcripts (Minamino and Macnab, 1999; Niehus et al., 2004; Tsang et al., 2015; Barker et al., 2016).

In Enterobacteriaceae as well as in Epsilon-proteobacteria, late FlhA-dependent regulatory events involve the anti-sigma factor FlgM, whereby the disruption of the flagellar secretion process directly impacts on late gene regulation (Josenhans et al., 2002; Rust et al., 2009; Chevance and Hughes, 2017). However, it is not known how flagellar structural proteins such as FlhA can contribute to flagellar type III secretion-independent regulation events of earlier expressed genes (class 2 flagellar genes). FlhA is known to harbor a critical patch of charged amino acid residues in a cytoplasmic loop which can influence its conformation (Erhardt et al., 2017). In *H. pylori*, the expression of intermediate (class 2 and class 3) flagellar genes is regulated by the cytoplasmic TCS FlgS-FlgR and its impact on σ54-dependent transcription (Niehus et al., 2004). The signal sensed by the cytoplasmic sensor histidine kinase FlgS and which initiates middle (class 2) flagellar gene expression is currently unknown. Recent studies indicated that FlhA of *H. pylori* directly interacts with cytoplasmic FlgS (Tsang et al., 2015). This protein-protein interaction might be of particular importance to the cytoplasmic sensory activity of FlgS and its downstream regulation via the response regulator FlgR, and may subsequently serve to maintain a functional flagellar assembly (Tsang et al., 2015). The same group has also demonstrated that the N-terminal domain of FlhA was sufficient for the σ54-dependent expression of the middle flagellar genes (Tsang et al., 2013), while it was not sufficient to mediate flagellar assembly and secretion. Since the direct interaction between FlgS and FlhA did not cause downstream signaling via FlgR phosphorylation in a reconstituted system *in vitro*, this protein-protein interaction may not be functional alone, but may be just one component of a larger, functional, protein complex involving other regulatory proteins (Tsang et al., 2015). The response regulator FlgR can act as a transcriptional activator without its cognate sensor kinase and is constitutively active in the absence of phosphorylation. Phosphorylation might therefore serve to modulate its activity or alter its range of transcriptional targets. Interestingly, the FlgS-FlgR pair do not only regulate motility genes but also metabolic functions (Niehus et al., 2004), which may provide a functional coupling between metabolic adaptation and motility. The mechanisms of function of FlgS/FlgR-like regulators in other bacterial species (Dasgupta et al., 2003; Jacobi et al., 2004; Boll and Hendrixson, 2013) remain yet to be clarified.

In summary, these novel interaction-dependent mechanisms of flagellar sensing indicate that flagellar hierarchy is not only governed by transcriptional regulators involving transmembrane TCS and transport-dependent anti-sigma factors, but also by an intracellular, TCS-dependent sensing mechanism which is not acting alone, but requires auxiliary proteins. These can be structural proteins such as FlhA, but may also involve larger protein complexes. The mechanisms of non-canonical sensing and protein interactions in flagellar regulation in various species need to be investigated in more detail.

## Modulation of Motility and Taxis by Co-Regulatory Protein-Protein Interactions and Conformational Changes

Chemotaxis sensors can be transmembrane or soluble dimeric sensors, which form arrays of trimers of dimers (Briegel et al., 2014; Mauriello et al., 2018) and sense different environmental and internal cues. By interacting with the chemotaxis TCS formed by CheA and CheY, they feed sensory information to the basal body of the flagellar organelle (Porter et al., 2011; Parkinson et al., 2015). Several studies in different bacterial species provided preliminary data that taxis systems and flagella can sense protein activities or changes in protein-protein interactions, reviewed in Anderson et al. (2010), and also further outlined below. This may be of particular importance for motility functions, since they are required to provide a rapid response to changing environmental conditions, by modifications of either flagellar rotational speed or the direction of rotation (Baron et al., 2017; Koganitsky et al., 2019; Nieto et al., 2019), which are both important for the directionality of the biased random walk. Flagellar speed is set at the rotary motor and its interacting flagellar basal body proteins (Nesper et al., 2017). The direction of flagella rotation is influenced by receptor proteins (methyl-accepting proteins [MCP]), which transmit signals to the flagellar base proteins (FliM) via the kinase CheA (Muok et al., 2019a) and the taxis response regulator protein CheY (Figure 1) (Baker et al., 2006; Di Paolo et al., 2016; Nishikino et al., 2018; Mukherjee et al., 2019; Ward et al., 2019). Therefore, signals feeding into flagellar rotational speed and direction intersect at the flagellar basal body. Several independent studies discussed below reported that proteins of the electron transport chain directly affect motility and taxis. Earlier it was demonstrated that a direct interaction of the bacterial fumarate reductase with the flagellar switch apparatus in *E. coli*, which constitutes a regulatory factor for flagellar rotation, depends on fumarate reductase enzymatic activity (Barak et al., 1996; Cohen-Ben-Lulu et al., 2008). Later, it was also reported that both the F_0_F_1_ ATP synthase and NADH-ubiquinone oxidoreductase interact with flagellar basal body proteins in *E. coli* (Zarbiv et al., 2012). Furthermore, microscopy studies demonstrated that respiratory chain components of *E. coli* and *B. subtilis* arrange in mobile or dynamic aggregations, which are distributed over the whole cell membrane without a preference for the poles or permanent co-localization with the chemotaxis clusters at this site (Johnson et al., 2004; Lenn et al., 2008; Llorente-Garcia et al., 2014). It can be hypothesized that respiratory chain proteins co-localize with chemotaxis clusters or flagella only temporarily and in a dynamic manner, which might then be instrumental for regulatory processes impacting on speed or direction of flagellar rotation.

In fact, besides modes of bacterial chemotaxis sensing which is based on direct binding of small molecule ligands to periplasmic receptor domains of the MCPs, an alternative sensing mechanism, termed *energy taxis*, has been observed to be widespread in diverse bacterial and archaeal species (Glagolev, 1980; Taylor et al., 1999; Schweinitzer et al., 2008; Alexandre, 2010). Energy taxis can be defined as a metabolism-dependent sensing by intracellular sensors, which includes the integration of multiple cues and signals derived from environmental conditions related to the intra-bacterial energy state and possibly involving protein activity (Taylor et al., 1999; Schweinitzer and Josenhans, 2010). However, distinct mechanisms of energy sensing have only been reported so far for a handful of designated receptor proteins, which are not canonical transmembrane receptors, but are placed in the cytoplasm (Packer and Armitage, 2000a, b; Muok et al., 2019b). One family of intracellular soluble receptors, which respond to oxidative conditions and oxygen and were previously suspected to sense energy indirectly, are in fact activated by an auxiliary sensory subunit protein, ODP, which can directly bind iron and oxygen (Muok et al., 2019b). ODP directly interacts conformation-dependently with its cognate receptors. However, in the absence of a dedicated auxiliary sensory unit protein, other mechanisms related to protein activity sensing have been proposed for several receptor proteins as summarized below.

The chemotaxis model organism *E. coli* was shown to employ two energy sensors, Aer and Tsr (Rebbapragada et al., 1997; Edwards et al., 2006). Aer, initially characterized as an aerotaxis or oxygen taxis sensor carrying a PAS domain, was demonstrated to respond to the intra-bacterial redox potential associated with the electron transport chain. In-depth studies into the *E. coli* Aer sensing mechanism suggest that a close, likely direct, physical association of Aer and the respiratory enzyme NADH dehydrogenase exists (Edwards et al., 2006). Suggestions on the molecular mechanism have taken into consideration a sensing of electrons from the electron transport chain to Aer via its non-covalently bound FAD cofactor (Rebbapragada et al., 1997; Garcia et al., 2016; Samanta et al., 2016), and a direct interaction of Aer with an electron transport chain enzyme (Edwards et al., 2006). Such an interaction could deliver information on the intracellular redox potential to Aer. Currently, there is no evidence for direct ligand binding other than FAD by Aer or direct protein-protein interactions. Further studies are needed to address the hypothesis of Aer activity sensing.

More evidence for a strong connection between membrane processes, nutrient acquisition, metabolism, and motility regulation comes from carbohydrate uptake systems at the bacterial membrane. Carbohydrate uptake by the phosphotransferase system (PTS) is a central metabolic function for carbohydrate uptake in many bacteria that also has diverse roles in regulation, including motility and taxis (Deutscher, 2008; Joyet et al., 2013; Deutscher et al., 2014).

The PTS is one of the early examples of *protein activity sensing* by the activity of a membrane transport system, which is metabolically active and at the same time relays information to transcriptional regulators. In many Proteobacteria, also a truncated, secondary PTS exists (PTSNtr), which only acts in regulation. In *E. coli*, PTSNtr interacts with the potassium transporter TrkA and the sensor kinase KdpD, influencing the activity and expression of these and other target proteins (Luttmann et al., 2015). In *R. eutropha*, EIIA(Ntr) interacts with the bifunctional ppGpp synthase/hydrolase SpoT1, a key enzyme and regulator of the stringent response (Karstens et al., 2014). The EIIA(Ntr) enzyme of this truncated system was thereby able to regulate different metabolic pathways and to transfer nutritional information to the regulatory systems of the bacteria. Other PTS components such as the small heat-stable phospho-carrier protein HPr are also able to bind transcriptional regulators, such as the stationary-phase anti-sigma factor Rsd, which regulates SigmaD activity in *E. coli*, but the downstream outcome of this interaction is less clear (Neira et al., 2018).

The PTS system, among other regulatory functions, also delivers signals to the chemotaxis cascade (Neumann et al., 2012) which then regulates chemotaxis. This was underpinned in a more recent study, when direct protein-protein interactions of PTS components with the taxis TCS were studied and confirmed by positive FRET signals between a CheZ and CheY FRET pair in *E. coli* (Somavanshi et al., 2016). In these assays, it was demonstrated that the integrated output of the complete PTS network is transmitted linearly to the chemotaxis pathway, in stark contrast to the amplification of conventional chemotactic stimuli (Somavanshi et al., 2016).

Earlier, RecA, the major bacterial recombinase which mediates homologous recombination of DNA, has been shown to play a role in chemotactic and swarming phenotypes and polar localization of a chemoreceptor in *Salmonella enterica* serovar Typhimurium (Mayola et al., 2014). RecA was also found to interact directly with the taxis adaptor protein CheW. In a follow-up study, the same group demonstrated that the inhibition of bacterial surface swarming was due to the net increase of RecA over the taxis sensor kinase CheA in stressed cultures which mounted an SOS response (Irazoki et al., 2016). However, only the presence and increase in protein amounts of RecA, and not necessarily its activation, were sufficient to stop swarming ability. This phenotype seems to be directly related to the extent of polar localization of the chemotaxis machinery, but the exact mechanism remains to be clarified (Irazoki et al., 2016).

In another model bacterium with four taxis sensors, *H. pylori*, the activity of the electron transport chain as a marker of metabolic sufficiency has been proposed to be crucial for energy-tactic behavior by the non-transmembrane taxis receptor TlpD, although it is not clear whether protein activity sensing might directly contribute to these functions (Schweinitzer et al., 2008; Collins et al., 2016). The soluble intracytoplasmic taxis sensor TlpD mediates energy taxis (Schweinitzer et al., 2008), which has been demonstrated as repellent taxis away from conditions of reduced respiratory chain activity, such as that induced by specific inhibitors (Schweinitzer et al., 2008) or redox stress (Collins et al., 2016). Several of the respiratory chain proteins in all bacteria contain iron-sulfur clusters which are redox-sensitive. In *H. pylori*, some of those are connected to sensing performed by TlpD (Behrens et al., 2016). The authors identified several novel intracellular protein interaction partners of TlpD by mass spectrometry, including the iron-sulfur cluster-containing enzyme aconitase (AcnB) (Austin et al., 2015; Behrens et al., 2016). Besides the metabolic enzyme AcnB and the chemotaxis histidine kinase CheAY2, catalase KatA was detected as direct physical interactor of TlpD. Additionally, AcnB and KatA were shown to play a role in the subcellular localization of TlpD (Behrens et al., 2016). The subcellular localization of TlpD can vary between the formation of polar foci, and a cytoplasmic diffuse localized portion, dependent on external nutrient availability and stress conditions (Behrens et al., 2016). However, despite a clear involvement of the respiratory chain in this energy sensing process (Schweinitzer et al., 2008), no evidence has been obtained yet that electron transport chain components interact directly with the chemotaxis or flagella machinery in *H. pylori*.

Conversely, TlpD had an effect on transcriptional regulation, impacting the expression of numerous genes involved in redox homeostasis and metabolism (Behrens et al., 2016). Therefore, it has been hypothesized that TlpD could serve as a linker between the taxis response and metabolic regulatory responses, possibly via the bifunctional regulatory protein AcnB (Austin et al., 2015; Behrens et al., 2016) (see next chapter for AcnB regulatory function). This direct link might be required to cope with energy limitation conditions, thus providing an immediate mechanism to improve the intracellular state of the cell. Furthermore, TlpD integrates responses of the other *H. pylori* taxis sensors (TlpA, TlpB, and TlpC), because it can initiate a dual response not only on the level of motility in the short-term but also by influencing metabolic gene regulation in the medium temporal range.

Taken together, emerging evidence shows the presence of a functional coupling, either by direct interaction or regulatory effects of metabolic enzymes, between electron transport chain components and other transport systems to regulators of metabolic functions, motility and flagella, including the bacterial taxis systems. We argue that this link is crucial in order to connect the energy-consuming functions of the flagellar apparatus to the sum of metabolic activities providing the necessary energy. This link might have been selected during evolution to provide useful feedback responses from metabolism to motility and taxis and vice-versa, in order to adjust motility functions, such as flagellar rotation, number of flagella, or the functionality and amount of chemotaxis receptors to the levels in energy and metabolic activity. These connections underline how important it is for bacteria to efficiently transmit and integrate information on the intracellular energy and nutritional levels into their sensory circuitry. Although the function and evolutionary aspects of the activity sensing processes related to motility are not entirely clear, they definitely highlight the importance to better understand the mechanisms behind the sensing of the intracellular energy state, which may depend on protein activities and protein-protein interactions.

## Important Moonlighting Proteins in Motility and Metabolism

A discussion about protein activity sensing in metabolism and motility also requires the mention of the term moonlighting proteins. The term was coined about two decades ago (Jeffery, 1999), to describe proteins which carry out several (at least two) physiological functions, which frequently include protein or DNA binding at the same time as they undergo conformational change, which leads to the loss of another function or activity such as a catalytic one (Jeffery, 2019). Some of these proteins can perform their two roles in parallel or shift gradually between two functions, depending on the environmental and intracellular conditions. Up to now, more than 300 moonlighting proteins have been described in different classes of protein functions, for instance, metabolic enzymes, transcription factors and receptors, comprising many eukaryotic and prokaryotic species (Jeffery, 2014). Since their enzyme activity is closely connected to the surrounding conditions, they represent another layer in the regulatory network within a cell. Among the many moonlighting enzymes and proteins with dual, alternating functions, there are numerous examples which moonlight as important regulators or co-regulators of metabolism in bacteria, but relatively few for motility functions. Indeed, we have recognized some novel moonlighting proteins which have a second function as sensors already in the above paragraphs, and we will highlight here a few others which are specifically involved in motility and metabolism.

One prominent and well-characterized example is the citrate cycle enzyme aconitase (AcnB). AcnB has been described as a moonlighting protein with dual functions in *E. coli*, *H. pylori* and other organisms including *Homo sapiens* (Tang et al., 2005; Austin and Maier, 2013; Jeffery, 2014). Several studies concordantly show that aconitases of various organisms can perform a biphasic switch between a metabolically active and a regulatory function by protein conformational changes (Kiley and Beinert, 2003; Tang et al., 2005; Austin and Maier, 2013; Lushchak et al., 2014; Austin et al., 2015). Under oxidative stress or iron depletion, which destroys the iron-sulfur clusters essential for its enzymatic activity, aconitase partially unfolds and can itself bind to particular mRNA sequences, resulting in either an increased or decreased mRNA stability and altered translation of the respective proteins (Lushchak et al., 2014). This ability to couple protein activity with posttranscriptional regulation constitutes a second mode of bacterial sensing-signaling of protein activities with conformational change. In *H. pylori*, aconitase-dependent regulation impacts on both metabolic and motility functions (Austin et al., 2015). By RNA mobility shift and proteomics, the authors of the latter study demonstrated that aconitase influences the expression of the intracellular flagellar regulator *flgR*/FlgR, the FlgRS-RpoN-FlgE cascade, and the metabolic proteins urease and hydrogenase. Aconitase also exerts an influence on flagellin gene expression and motility in *S. enterica* serovar Typhimurium (Tang et al., 2004). Concordantly, an aconitase mutant showed considerably lower protein levels of the main flagellin FliC. The authors found that this is indirectly mediated, most likely by a stabilizing effect of aconitase on mRNA of the protease gene *ftsH* (Tang et al., 2004).

In *Listeria monocytogenes*, which is only motile at lower temperatures due to its biphasic switch between environmental and host-associated lifestyle, GmaR, a bifunctional Glc-Nac transferase, moonlights in motility regulation under conditions of low temperature, when DegU-dependent transcription of its own gene is initiated (Shen et al., 2006). Upon production, GmaR binds the constitutively expressed motility repressor MogR and relieves MogR repressor function on motility genes. At the same time, GmaR functionally acts as a glycosylation enzyme for the main flagellin FlaA, which it helps to produce. Upon a temperature shift to 37°C, GmaR undergoes a conformational change which abolishes its MogR binding and anti-repressor function (Kamp and Higgins, 2011).

Numerous examples of mostly metabolic proteins moonlighting as transcriptional or posttranscriptional co-regulators have been reported (Commichau and Stülke, 2015; Monahan and Harry, 2016). In the following, we would like to highlight just a few of those which are so far known exclusively for broadly regulating core metabolic functions. One important moonlighting protein involved in metabolic regulation is PutA of *Salmonella*, which, on one hand, functions as a proline dehydrogenase, converting proline to glutamate for metabolic purposes, and on the other, as a direct transcriptional repressor of the *put* operon involved in amino acid conversion (Liu et al., 2017). Similarly, BirA of *E. coli* harbors enzymatic activity as a biotin synthetase and moonlights as a transcriptional repressor of the *bio* operon (Barker and Campbell, 1981). In the *B. subtilis* glutamine pathway, the enzyme glutamine synthetase, termed an activity sensor for nitrogen availability (Wray et al., 2001; Mirouze et al., 2015), binds to the transcription factor TnrA, a global regulator of transcription, and prevents TnrA from binding to DNA (Wray et al., 2001), which influences multiple metabolic pathways (Mirouze et al., 2015; Randazzo et al., 2017). Also in the glutamate pathway, glutamate dehydrogenase interacts directly with GltC, a LysR-type transcription factor, to influence its transcription-enhancing activity (Gunka et al., 2010; Stannek et al., 2015). Additional examples of mostly metabolic proteins moonlighting as transcriptional or posttranscriptional co-regulators have been reported (Commichau and Stülke, 2015; Monahan and Harry, 2016).

In summary, there are many examples which link the maintenance of physiological and metabolic functions to activity sensing mechanisms. Metabolic functions and the control of influx of metabolic substrates are an important source of protein co-factors and sensory proteins necessary for the regulation of intracellular metabolism and other important functions such as motility.

## Integration of Bacterial Protein Activity Sensing Into Regulatory Circuitry and Environmental Adaptation

While bacteria have evolved highly coordinated regulatory systems, it is unclear why protein activity sensing is required or how it is integrated into the regulatory circuitry. Signal integration can work by varying protein expression levels, their activity, conformation and the subcellular localization of the sensory cofactors or the activity-sensing components themselves. Several examples explained above highlight that protein activity sensing in combination with expression levels and feedback mechanisms helps to integrate information on metabolic activities, environmental conditions such as temperature, nutrient transport, oxygen and iron stress, in a direct and fast manner.

Alternative models of signal integration into circuits, which are emerging from recent studies in different bacterial species, include a dynamic protein localization with subcellular rearrangements upon different signals, not only for transmembrane receptors but also for other bacterial proteins (Borrok et al., 2008; Wu et al., 2011; Alberge et al., 2015; Hiremath et al., 2015). In particular, metabolic features in general seem to have a high potential to influence protein localization. For example, in *Vibrio cholerae*, one set of transmembrane chemotaxis components localized either at the pole or diffuse in the cell depending on the presence and absence of oxygen which can be used as electron acceptor (Hiremath et al., 2015). Recently, in *E. coli* the nitrate reductase, one component of the electron transport chain which is necessary for anaerobic respiration, was also reported to localize dynamically upon switches between aerobic or anaerobic conditions (Alberge et al., 2015). Only few data are available on soluble cytoplasmic receptors which might regulate motility. One important example is the soluble taxis receptor AerC from *A. brasilense*, which dynamically localizes to the cell poles depending on the levels of oxygen, which is an important factor to guide the bacteria and contribute to their metabolic activity (Xie et al., 2010).

In order for bacteria to thrive in different habitats, efficiency in energy sensing and the integration of multiple signals are essential. For pathogenic and non-pathogenic bacteria, the regulatory properties and survival strategies have been evolved to cope with complex and variable environments. While in the early days of pathogen research, researchers focused on the effect of virulence factors in host damage, recently the combination of bacterial traits is viewed more in the context of optimizing the metabolic situation inside the host niche to the benefit of specific bacteria (Winter et al., 2010; Rivera-Chavez et al., 2013, 2016; Eisenreich et al., 2015). This requires multiple modes of signal integration and regulatory crosstalk, which might involve the direct and indirect sensing of environment- or host-derived compounds and metabolites (Sule et al., 2017; Lopes and Sourjik, 2018) as well as metabolic protein activities which feed into sensing. In addition to taxis receptors which perform regulatory functions, some organisms might also dedicate entire chemotaxis systems to perform exclusively regulatory functions benefiting specific lifestyles such as bacterial biofilms or host-associated biology (García-Fontana et al., 2013; Xu et al., 2017). For instance, in the enteropathogen *V. cholerae*, there are three different operons (cluster I, II, and II) encoding for chemotactic-related genes (Ringgaard et al., 2018). In *V. cholerae*, genes from cluster II encode proteins responsible for chemotactic behavior in most environmental conditions. Meanwhile, cytoplasmic receptors associated with proteins encoded by genes from cluster I are specifically induced under low oxygen conditions (Hiremath et al., 2015), leading to the suggestion that these particular receptors and its associated proteins are responsible for chemotactic activity and survival in the host intestine (Hiremath et al., 2015; Briegel et al., 2016). Moreover, it has been observed that cluster III proteins are observable in microscopy studies during stationary phase only (Ringgaard et al., 2015). Other studies have shown that several chemosensory systems have outputs other than mediating a chemotactic response (Kirby, 2009; Wuichet and Zhulin, 2010), and instead regulate alternative functions, such as type IV-pili-mediated motility or the modulation of intracellular levels of secondary messengers (Hickman et al., 2005; Zusman et al., 2007; Wuichet and Zhulin, 2010).

## Conclusion and Future Directions

Numerous examples now illustrate the role of non-regulator proteins with important primary functions in membrane transport or metabolism as cofactors in central regulatory processes, for example those governing bacterial metabolism, taxis and motility. However, some of the important mechanisms of their sensory and regulatory functionality, in the context of their structure and possible modifications, remain unknown. More examples of complex sensory perception and signal integration by activity sensing and protein-protein interactions are to be discovered in all bacterial taxons. Further research is required to decipher the mechanisms behind temporal and spatial protein activities and localization dynamics, and how those feed into their activities as sensors, cofactors or transmitters of signals. The value of such functions can also be appreciated by mathematically modeling their influence on the activity kinetics of sensory systems and their output. A greater understanding of these processes will be beneficial to the study of bacterial sensing, inter-species communication and their proliferative capacities in the context of hosts and other relevant environments.

## Author Contributions

All authors wrote the manuscript jointly. AA and CJ revised the manuscript critically for timely content and structure. CJ developed the initial concept of the article and acquired funding. All authors edited, revised, and approved the final manuscript.

## Conflict of Interest

The authors declare that the research was conducted in the absence of any commercial or financial relationships that could be construed as a potential conflict of interest.

## References

[B1] Alberge F. Espinosa L. Seduk F. Sylvi L. Toci R. Walburger A. (2015). Dynamic subcellular localization of a respiratory complex controls bacterial respiration. *eLife* 4:e05357 10.7554/eLife.05357 PMC4466248 26077726

[B2] Alexandre G. (2010). Coupling metabolism and chemotaxis-dependent behaviours by energy taxis receptors. *Microbiology* 156 2283–2293. 10.1099/mic.0.039214-0 20558508

[B3] Anderson J. K. Smith T. G. Hoover T. R. (2010). Sense and sensibility: flagellum-mediated gene regulation. *Trends Microbiol.* 18 30–37. 10.1016/j.tim.2009.11.001 19942438 PMC2818477

[B4] Armitage J. P. (1997). Behavioural responses of bacteria to light and oxygen. *Arch. Microbiol.* 168 249–261. 10.1007/s002030050496 9297461

[B5] Ashby M. K. (2004). Survey of the number of two-component response regulator genes in the complete and annotated genome sequences of prokaryotes. *FEMS Microbiol. Lett.* 231 277–281. 10.1016/S0378-1097(04)00004-7 14987775

[B6] Austin C. M. Maier R. J. (2013). Aconitase-mediated posttranscriptional regulation of *Helicobacter pylori* peptidoglycan deacetylase. *J. Bacteriol.* 195 5316–5322. 10.1128/JB.00720-13 24056106 PMC3837956

[B7] Austin C. M. Wang G. Maier R. J. (2015). Aconitase functions as a pleiotropic posttranscriptional regulator in *Helicobacter pylori*. *J. Bacteriol.* 197 3076–3086. 10.1128/JB.00529-15 26170414 PMC4560275

[B8] Baker M. D. Wolanin P. M. Stock J. B. (2006). Systems biology of bacterial chemotaxis. *Curr. Opin. Microbiol.* 9 187–192. 10.1016/j.mib.2006.02.007 16529985

[B9] Bange G. Petzold G. Wild K. Parlitz R. O. Sinning I. (2007). The crystal structure of the third signal-recognition particle GTPase FlhF reveals a homodimer with bound GTP. *Proc. Natl. Acad. Sci. U.S.A.* 104 13621–13625. 10.1073/pnas.0702570104 17699634 PMC1959431

[B10] Barak R. Giebel I. Eisenbach M. (1996). The specificity of fumarate as a switching factor of the bacterial flagellar motor. *Mol. Microbiol.* 19 139–144. 10.1046/j.1365-2958.1996.365889.x 8821943

[B11] Barker C. S. Inoue T. Meshcheryakova I. V. Kitanobo S. Samatey F. A. (2016). Function of the conserved FHIPEP domain of the flagellar type III export apparatus, protein FlhA. *Mol. Microbiol.* 100 278–288. 10.1111/mmi.13315 26691662

[B12] Barker D. F. Campbell A. M. (1981). Genetic and biochemical characterization of the *birA* gene and its product: evidence for a direct role of biotin holoenzyme synthetase in repression of the biotin operon in *Escherichia coli*. *J. Mol. Biol.* 146 469–492. 10.1016/0022-2836(81)90043-7 6456358

[B13] Baron S. Afanzar O. Eisenbach M. (2017). Methylation-independent adaptation in chemotaxis of *Escherichia coli* involves acetylation-dependent speed adaptation. *FEBS Lett.* 591 331–337. 10.1002/1873-3468.12537 27995613

[B14] Behrens W. Schweinitzer T. Mcmurry J. L. Loewen P. C. Buettner F. F. Menz S. (2016). Localisation and protein-protein interactions of the *Helicobacter pylori* taxis sensor TlpD and their connection to metabolic functions. *Sci. Rep.* 6:23582. 10.1038/srep23582 27045738 PMC4820699

[B15] Bernard R. Guiseppi A. Chippaux M. Foglino M. Denizot F. (2007). Resistance to bacitracin in *Bacillus subtilis*: unexpected requirement of the BceAB ABC transporter in the control of expression of its own structural genes. *J. Bacteriol.* 189 8636–8642. 10.1128/JB.01132-07 17905982 PMC2168949

[B16] Boll J. M. Hendrixson D. R. (2013). A regulatory checkpoint during flagellar biogenesis in *Campylobacter jejuni* initiates signal transduction to activate transcription of flagellar genes. *mBio* 4:e00432-13. 10.1128/mBio.00432-13 24003178 PMC3760246

[B17] Borrok M. J. Kolonko E. M. Kiessling L. L. (2008). Chemical probes of bacterial signal transduction reveal that repellents stabilize and attractants destabilize the chemoreceptor array. *ACS Chem. Biol.* 3 101–109. 10.1021/cb700211s 18278851

[B18] Bourret R. B. Silversmith R. E. (2010). Two-component signal transduction. *Curr. Opin. Microbiol.* 13 113–115. 10.1016/j.mib.2010.02.003 20219418 PMC2847671

[B19] Brameyer S. Rösch T. C. El Andari J. Hoyer E. Schwarz J. Graumann P. L. (2019). DNA-binding directs the localization of a membrane-integrated receptor of the ToxR family. *Commun. Biol.* 2:4. 10.1038/s42003-018-0248-7 30740540 PMC6320335

[B20] Briegel A. Ladinsky M. S. Oikonomou C. Jones C. W. Harris M. J. Fowler D. J. (2014). Structure of bacterial cytoplasmic chemoreceptor arrays and implications for chemotactic signaling. *eLife* 3:e02151. 10.7554/eLife.02151 24668172 PMC3964821

[B21] Briegel A. Ortega D. R. Mann P. Kjaer A. Ringgaard S. Jensen G. J. (2016). Chemotaxis cluster 1 proteins form cytoplasmic arrays in *Vibrio cholerae* and are stabilized by a double signaling domain receptor DosM. *Proc. Natl. Acad. Sci. U.S.A.* 113 10412–10417. 10.1073/pnas.1604693113 27573843 PMC5027440

[B22] Buchner S. Schlundt A. Lassak J. Sattler M. Jung K. (2015). Structural and functional analysis of the signal-transducing linker in the pH-responsive one-component system CadC of *Escherichia coli*. *J. Mol. Biol.* 427 2548–2561. 10.1016/j.jmb.2015.05.001 25979249

[B23] Buelow D. R. Raivio T. L. (2010). Three (and more) component regulatory systems–auxiliary regulators of bacterial histidine kinases. *Mol. Microbiol.* 75 547–566. 10.1111/j.1365-2958.2009.06982.x 19943903

[B24] Buhrke T. Lenz O. Porthun A. Friedrich B. (2004). The H2-sensing complex of *Ralstonia eutropha*: interaction between a regulatory [NiFe] hydrogenase and a histidine protein kinase. *Mol. Microbiol.* 51 1677–1689. 10.1111/j.1365-2958.2003.03933.x 15009894

[B25] Capra E. J. Laub M. T. (2012). Evolution of two-component signal transduction systems. *Annu. Rev. Microbiol.* 66 325–347. 10.1146/annurev-micro-092611-150039 22746333 PMC4097194

[B26] Chevance F. F. Hughes K. T. (2017). Case for the genetic code as a triplet of triplets. *Proc. Natl. Acad. Sci. U.S.A.* 114 4745–4750. 10.1073/pnas.1614896114 28416671 PMC5422812

[B27] Clemens R. Zaschke-Kriesche J. Khosa S. Smits S. H. J. (2018). Insight into two ABC transporter families involved in lantibiotic resistance. *Front. Mol. Biosci.* 4:91. 10.3389/fmolb.2017.00091 29404338 PMC5786555

[B28] Cohen-Ben-Lulu G. N. Francis N. R. Shimoni E. Noy D. Davidov Y. Prasad K. (2008). The bacterial flagellar switch complex is getting more complex. *EMBO J.* 27 1134–1144. 10.1038/emboj.2008.48 18337747 PMC2323253

[B29] Collins K. D. Andermann T. M. Draper J. Sanders L. Williams S. M. Araghi C. (2016). The *Helicobacter pylori* CZB cytoplasmic chemoreceptor TlpD forms an autonomous polar chemotaxis signaling complex that mediates a tactic response to oxidative stress. *J. Bacteriol.* 198 1563–1575. 10.1128/JB.00071-16 27002127 PMC4959281

[B30] Commichau F. M. Stülke J. (2015). Trigger enzymes: coordination of metabolism and virulence gene expression. *Microbiol. Spectr.* 3 105–127. 10.1128/microbiolspec.MBP-0010-2014 26350309

[B31] Coumes-Florens S. Brochier-Armanet C. Guiseppi A. Denizot F. Foglino M. (2011). A new highly conserved antibiotic sensing/resistance pathway in firmicutes involves an ABC transporter interplaying with a signal transduction system. *PLoS One* 6:e15951. 10.1371/journal.pone.0015951 21283517 PMC3023708

[B32] Dasgupta N. Wolfgang M. C. Goodman A. L. Arora S. K. Jyot J. Lory S. (2003). A four-tiered transcriptional regulatory circuit controls flagellar biogenesis in *Pseudomonas aeruginosa*. *Mol. Microbiol.* 50 809–824. 10.1046/j.1365-2958.2003.03740.x 14617143

[B33] Desai S. K. Kenney L. J. (2017). To approximately P or Not to approximately P? Non-canonical activation by two-component response regulators. *Mol. Microbiol.* 103 203–213. 10.1111/mmi.13532 27656860 PMC5218973

[B34] Deutscher J. (2008). The mechanisms of carbon catabolite repression in bacteria. *Curr. Opin. Microbiol.* 11 87–93. 10.1016/j.mib.2008.02.007 18359269

[B35] Deutscher J. Ake F. M. Derkaoui M. Zebre A. C. Cao T. N. Bouraoui H. (2014). The bacterial phosphoenolpyruvate:carbohydrate phosphotransferase system: regulation by protein phosphorylation and phosphorylation-dependent protein-protein interactions. *Microbiol. Mol. Biol. Rev.* 78 231–256. 10.1128/MMBR.00001-14 24847021 PMC4054256

[B36] Di Paolo D. Afanzar O. Armitage J. P. Berry R. M. (2016). Single-molecule imaging of electroporated dye-labelled CheY in live *Escherichia coli*. *Philos. Trans. R. Soc. Lond B Biol. Sci.* 371 1–13. 10.1098/rstb.2015.0492 27672145 PMC5052738

[B37] Dintner S. Heermann R. Fang C. Jung K. Gebhard S. (2014). A sensory complex consisting of an ATP-binding cassette transporter and a two-component regulatory system controls bacitracin resistance in *Bacillus subtilis*. *J. Biol. Chem.* 289 27899–27910. 10.1074/jbc.M114.596221 25118291 PMC4183823

[B38] Dintner S. Staron A. Berchtold E. Petri T. Mascher T. Gebhard S. (2011). Coevolution of ABC transporters and two-component regulatory systems as resistance modules against antimicrobial peptides in Firmicutes Bacteria. *J. Bacteriol.* 193 3851–3862. 10.1128/JB.05175-11 21665979 PMC3147537

[B39] Dufour Y. S. Fu X. Hernandez-Nunez L. Emonet T. (2014). Limits of feedback control in bacterial chemotaxis. *PLoS Comput. Biol.* 10:e1003694. 10.1371/journal.pcbi.1003694 24967937 PMC4072517

[B40] Edwards J. C. Johnson M. S. Taylor B. L. (2006). Differentiation between electron transport sensing and proton motive force sensing by the Aer and Tsr receptors for aerotaxis. *Mol. Microbiol.* 62 823–837. 10.1111/j.1365-2958.2006.05411.x 16995896 PMC1858650

[B41] Eisenreich W. Heesemann J. Rudel T. Goebel W. (2015). Metabolic adaptations of intracellullar bacterial pathogens and their mammalian host cells during infection (“pathometabolism”). *Microbiol. Spectr.* 3 27–58. 10.1128/microbiolspec.MBP-0002-2014 26185075

[B42] Erhardt M. Wheatley P. Kim E. A. Hirano T. Zhang Y. Sarkar M. K. (2017). Mechanism of type-III protein secretion: regulation of FlhA conformation by a functionally critical charged-residue cluster. *Mol. Microbiol.* 104 234–249. 10.1111/mmi.13623 28106310 PMC5380474

[B43] Fiebig A. Herrou J. Willett J. Crosson S. (2015). General stress signaling in the alphaproteobacteria. *Annu. Rev. Genet.* 49 603–625. 10.1146/annurev-genet-112414-054813 26442844 PMC4710059

[B44] Francis V. I. Porter S. L. (2019). Multikinase networks: two-component signaling networks integrating multiple stimuli. *Annu. Rev. Microbiol.* 73 199–223. 10.1146/annurev-micro-020518-115846 31112439

[B45] Fritz G. Dintner S. Treichel N. S. Radeck J. Gerland U. Mascher T. (2015). A new way of sensing: need-based activation of antibiotic resistance by a flux-sensing mechanism. *mBio* 6:e00975. 10.1128/mBio.00975-15 26199330 PMC4513084

[B46] Fritz G. Koller C. Burdack K. Tetsch L. Haneburger I. Jung K. (2009). Induction kinetics of a conditional pH stress response system in *Escherichia coli*. *J. Mol. Biol.* 393 272–286. 10.1016/j.jmb.2009.08.037 19703467

[B47] Galperin M. Y. (2018). What bacteria want. *Environ. Microbiol.* 20 4221–4229. 10.1111/1462-2920.14398 30187651 PMC7020242

[B48] Gao R. Stock A. M. (2009). Biological insights from structures of two-component proteins. *Annu. Rev. Microbiol.* 63 133–154. 10.1146/annurev.micro.091208.073214 19575571 PMC3645274

[B49] Garcia D. Watts K. J. Johnson M. S. Taylor B. L. (2016). Delineating PAS-HAMP interaction surfaces and signalling-associated changes in the aerotaxis receptor Aer. *Mol. Microbiol.* 100 156–172. 10.1111/mmi.13308 26713609 PMC5156559

[B50] García-Fontana C. Reyes-Darias J. A. Muñoz-Martínez F. Alfonso C. Morel B. Ramos J. L. (2013). High specificity in CheR methyltransferase function CheR2 of *Pseudomonas putida* is essential for chemotaxis, whereas CheR1 is involved in biofilm formation. *J. Biol. Chem.* 288 18987–18999. 10.1074/jbc.M113.472605 23677992 PMC3696673

[B51] Gardner S. G. Johns K. D. Tanner R. Mccleary W. R. (2014). The PhoU protein from *Escherichia coli* interacts with PhoR, PstB, and metals to form a phosphate-signaling complex at the membrane. *J. Bacteriol.* 196 1741–1752. 10.1128/JB.00029-14 24563032 PMC3993317

[B52] Gebhard S. (2012). ABC transporters of antimicrobial peptides in Firmicutes bacteria - phylogeny, function and regulation. *Mol. Microbiol.* 86 1295–1317. 10.1111/mmi.12078 23106164

[B53] Gebhard S. Mascher T. (2011). Antimicrobial peptide sensing and detoxification modules: unravelling the regulatory circuitry of *Staphylococcus aureus*. *Mol. Microbiol.* 81 581–587. 10.1111/j.1365-2958.2011.07747.x 21696467

[B54] Glagolev A. N. (1980). Reception of the energy level in bacterial taxis. *J. Theor. Biol.* 82 171–185. 10.1016/0022-5193(80)90097-1 6246312

[B55] Graf S. Schmieden D. Tschauner K. Hunke S. Unden G. (2014). The sensor kinase DctS forms a tripartite sensor unit with DctB and DctA for sensing C4-dicarboxylates in *Bacillus subtilis*. *J. Bacteriol.* 196 1084–1093. 10.1128/JB.01154-13 24375102 PMC3957698

[B56] Groeneveld M. Weme R. G. Duurkens R. H. Slotboom D. J. (2010). Biochemical characterization of the C4-dicarboxylate transporter DctA from *Bacillus subtilis*. *J. Bacteriol.* 192 2900–2907. 10.1128/JB.00136-10 20363944 PMC2876488

[B57] Groisman E. A. (2016). Feedback control of two-component regulatory systems. *Annu. Rev. Microbiol.* 70 103–124. 10.1146/annurev-micro-102215-095331 27607549 PMC8380452

[B58] Gunka K. Newman J. A. Commichau F. M. Herzberg C. Rodrigues C. Hewitt L. (2010). Functional dissection of a trigger enzyme: mutations of the *Bacillus subtilis* glutamate dehydrogenase RocG that affect differentially its catalytic activity and regulatory properties. *J. Mol. Biol.* 400 815–827. 10.1016/j.jmb.2010.05.055 20630473

[B59] Haneburger I. Eichinger A. Skerra A. Jung K. (2011). New insights into the signaling mechanism of the pH-responsive, membrane-integrated transcriptional activator CadC of *Escherichia coli*. *J. Biol. Chem.* 286 10681–10689. 10.1074/jbc.M110.196923 21216950 PMC3060519

[B60] Haneburger I. Fritz G. Jurkschat N. Tetsch L. Eichinger A. Skerra A. (2012). Deactivation of the *E. coli* pH stress sensor CadC by cadaverine. *J. Mol. Biol.* 424 15–27. 10.1016/j.jmb.2012.08.023 22999955

[B61] Hauf W. Schmid K. Gerhardt E. C. Huergo L. F. Forchhammer K. (2016). Interaction of the nitrogen regulatory protein GlnB (PII) with biotin carboxyl carrier protein (BCCP) Controls Acetyl-CoA Levels in the *Cyanobacterium Synechocystis* sp. PCC 6803. *Front. Microbiol.* 7:1700. 10.3389/fmicb.2016.01700 27833596 PMC5080355

[B62] Herrou J. Foreman R. Fiebig A. Crosson S. (2010). A structural model of anti-anti-sigma inhibition by a two-component receiver domain: the PhyR stress response regulator. *Mol. Microbiol.* 78 290–304. 10.1111/j.1365-2958.2010.07323.x 20735776 PMC2959141

[B63] Hervas A. B. Canosa I. Little R. Dixon R. Santero E. (2009). NtrC-dependent regulatory network for nitrogen assimilation in *Pseudomonas putida*. *J. Bacteriol.* 191 6123–6135. 10.1128/JB.00744-09 19648236 PMC2747892

[B64] Hickman J. W. Tifrea D. F. Harwood C. S. (2005). A chemosensory system that regulates biofilm formation through modulation of cyclic diguanylate levels. *Proc. Natl. Acad. Sci. U.S.A.* 102 14422–14427. 10.1073/pnas.0507170102 16186483 PMC1234902

[B65] Hiremath G. Hyakutake A. Yamamoto K. Ebisawa T. Nakamura T. Nishiyama S. (2015). Hypoxia-induced localization of chemotaxis-related signaling proteins in *Vibrio cholerae*. *Mol. Microbiol.* 95 780–790. 10.1111/mmi.12887 25420689

[B66] Hsieh Y. J. Wanner B. L. (2010). Global regulation by the seven-component Pi signaling system. *Curr. Opin. Microbiol.* 13 198–203. 10.1016/j.mib.2010.01.014 20171928 PMC2847643

[B67] Huergo L. F. Chandra G. Merrick M. (2013). P(II) signal transduction proteins: nitrogen regulation and beyond. *FEMS Microbiol. Rev.* 37 251–283. 10.1111/j.1574-6976.2012.00351.x 22861350

[B68] Inaba J. Thornton J. Huergo L. F. Monteiro R. A. Klassen G. Pedrosa Fde O. (2015). Mutational analysis of GlnB residues critical for NifA activation in *Azospirillum brasilense*. *Microbiol. Res.* 171 65–72. 10.1016/j.micres.2014.12.005 25644954

[B69] Irazoki O. Mayola A. Campoy S. Barbe J. (2016). SOS system induction inhibits the assembly of chemoreceptor signaling clusters in *Salmonella enterica*. *PLoS One* 11:e0146685. 10.1371/journal.pone.0146685 26784887 PMC4718596

[B70] Jacobi S. Schade R. Heuner K. (2004). Characterization of the alternative sigma factor sigma54 and the transcriptional regulator FleQ of *Legionella pneumophila*, which are both involved in the regulation cascade of flagellar gene expression. *J. Bacteriol.* 186:2540. 10.1128/JB.186.9.2540-2547.2004 15090493 PMC387802

[B71] Jeffery C. J. (1999). Moonlighting proteins. *Trends Biochem. Sci.* 24 8–11. 10.1016/s0968-0004(98)01335-8 10087914

[B72] Jeffery C. J. (2014). An introduction to protein moonlighting. *Biochem. Soc. Trans.* 42 1679–1683. 10.1042/BST20140226 25399589

[B73] Jeffery C. J. (2019). An enzyme in the test tube, and a transcription factor in the cell: moonlighting proteins and cellular factors that affect their behavior. *Protein Sci.* 28 1233–1238. 10.1002/pro.3645 31087733 PMC6566513

[B74] Johnson A. S. Van Horck S. Lewis P. J. (2004). Dynamic localization of membrane proteins in *Bacillus subtilis*. *Microbiology* 150 2815–2824. 10.1099/mic.0.27223-0 15347741

[B75] Josenhans C. Niehus E. Amersbach S. Hörster A. Betz C. Drescher B. (2002). Functional characterization of the antagonistic flagellar late regulators FliA and FlgM of *Helicobacter pylori* and their effects on the *H. pylori* transcriptome. *Mol. Microbiol.* 43 307–322. 10.1046/j.1365-2958.2002.02765.x 11985711

[B76] Joyet P. Bouraoui H. Ake F. M. Derkaoui M. Zebre A. C. Cao T. N. (2013). Transcription regulators controlled by interaction with enzyme IIB components of the phosphoenolpyruvate: sugar phosphotransferase system. *Biochim. Biophys. Acta* 1834 1415–1424. 10.1016/j.bbapap.2013.01.004 23318733

[B77] Jugder B. E. Chen Z. Ping D. T. Lebhar H. Welch J. Marquis C. P. (2015). An analysis of the changes in soluble hydrogenase and global gene expression in *Cupriavidus necator* (*Ralstonia eutropha*) H16 grown in heterotrophic diauxic batch culture. *Microb. Cell Fact.* 14 1–11. 10.1186/s12934-015-0226-4 25880663 PMC4377017

[B78] Kamp H. D. Higgins D. E. (2011). A protein thermometer controls temperature-dependent transcription of flagellar motility genes in *Listeria monocytogenes*. *PLoS Pathog.* 7:e1002153. 10.1371/journal.ppat.1002153 21829361 PMC3150276

[B79] Karstens K. Zschiedrich C. P. Bowien B. Stülke J. Görke B. (2014). Phosphotransferase protein EIIANtr interacts with SpoT, a key enzyme of the stringent response, in *Ralstonia eutropha* H16. *Microbiology* 160 711–722. 10.1099/mic.0.075226-0 24515609

[B80] Kazmierczak B. I. Hendrixson D. R. (2013). Spatial and numerical regulation of flagellar biosynthesis in polarly flagellated bacteria. *Mol. Microbiol.* 88 655–663. 10.1111/mmi.12221 23600726 PMC3654036

[B81] Kiley P. J. Beinert H. (2003). The role of Fe-S proteins in sensing and regulation in bacteria. *Curr. Opin. Microbiol.* 6 181–185. 10.1016/s1369-5274(03)00039-0 12732309

[B82] Kirby J. R. (2009). Chemotaxis-like regulatory systems: unique roles in diverse bacteria. *Annu. Rev. Microbiol.* 63 45–59. 10.1146/annurev.micro.091208.073221 19379070

[B83] Kleefeld A. Ackermann B. Bauer J. Krämer J. Unden G. (2009). The fumarate/succinate antiporter DcuB of *Escherichia coli* is a bifunctional protein with sites for regulation of DcuS-dependent gene expression. *J. Biol. Chem.* 284 265–275. 10.1074/jbc.M807856200 18957436

[B84] Kneuper H. Janausch I. G. Vijayan V. Zweckstetter M. Bock V. Griesinger C. (2005). The nature of the stimulus and of the fumarate binding site of the fumarate sensor DcuS of *Escherichia coli*. *J. Biol. Chem.* 280 20596–20603. 10.1074/jbc.M502015200 15781452

[B85] Koganitsky A. Tworowski D. Dadosh T. Cecchini G. Eisenbach M. (2019). A mechanism of modulating the direction of flagellar rotation in bacteria by fumarate and fumarate reductase. *J. Mol. Biol.* 431 3662–3676. 10.1016/j.jmb.2019.08.001 31412261 PMC6733631

[B86] Kondo S. Homma M. Kojima S. (2017). Analysis of the GTPase motif of FlhF in the control of the number and location of polar flagella in *Vibrio alginolyticus*. *Biophys. Physicobiol.* 14 173–181. 10.2142/biophysico.14.0_173 29362702 PMC5774409

[B87] Krell T. Lacal J. Busch A. Silva-Jimenez H. Guazzaroni M. E. Ramos J. L. (2010). Bacterial sensor kinases: diversity in the recognition of environmental signals. *Annu. Rev. Microbiol.* 64 539–559. 10.1146/annurev.micro.112408.134054 20825354

[B88] Lee Y. T. Wang M. C. (2019). The bacterivore’s solution: fight and flight to promote survival. *Dev. Cell* 49 7–9. 10.1016/j.devcel.2019.03.021 30965036

[B89] Lenn T. Leake M. C. Mullineaux C. W. (2008). Are *Escherichia coli* OXPHOS complexes concentrated in specialized zones within the plasma membrane? *Biochem. Soc. Trans.* 36 1032–1036. 10.1042/BST0361032 18793184

[B90] Lertsethtakarn P. Ottemann K. M. Hendrixson D. R. (2011). Motility and chemotaxis in *Campylobacter* and *Helicobacter*. *Annu. Rev. Microbiol.* 65 389–410. 10.1146/annurev-micro-090110-102908 21939377 PMC6238628

[B91] Lindner E. White S. H. (2014). Topology, dimerization, and stability of the single-span membrane protein CadC. *J. Mol. Biol.* 426 2942–2957. 10.1016/j.jmb.2014.06.006 24946151 PMC4126671

[B92] Liu L. K. Becker D. F. Tanner J. J. (2017). Structure, function, and mechanism of proline utilization A (PutA). *Arch. Biochem. Biophys.* 632 142–157. 10.1016/j.abb.2017.07.005 28712849 PMC5650515

[B93] Llorente-Garcia I. Lenn T. Erhardt H. Harriman O. L. Liu L. N. Robson A. (2014). Single-molecule in vivo imaging of bacterial respiratory complexes indicates delocalized oxidative phosphorylation. *Biochim. Biophys. Acta* 1837 811–824. 10.1016/j.bbabio.2014.01.020 24513194

[B94] Loh J. T. Gupta S. S. Friedman D. B. Krezel A. M. Cover T. L. (2010). Analysis of protein expression regulated by the *Helicobacter pylori* ArsRS two-component signal transduction system. *J. Bacteriol.* 192 2034–2043. 10.1128/JB.01703-08 20154125 PMC2849440

[B95] Lopes J. G. Sourjik V. (2018). Chemotaxis of *Escherichia coli* to major hormones and polyamines present in human gut. *ISME J.* 12 2736–2747. 10.1038/s41396-018-0227-5 29995838 PMC6194112

[B96] Löscher S. Gebler A. Stein M. Sanganas O. Buhrke T. Zebger I. (2010). Protein-protein complex formation affects the Ni-Fe and Fe-S centers in the H2-sensing regulatory hydrogenase from *Ralstonia eutropha* H16. *Chemphyschem* 11 1297–1306. 10.1002/cphc.200901007 20340124

[B97] Lushchak O. V. Piroddi M. Galli F. Lushchak V. I. (2014). Aconitase post-translational modification as a key in linkage between Krebs cycle, iron homeostasis, redox signaling, and metabolism of reactive oxygen species. *Redox Rep.* 19 8–15. 10.1179/1351000213Y.0000000073 24266943 PMC6837700

[B98] Luttmann D. Göpel Y. Görke B. (2015). Cross-talk between the canonical and the nitrogen-related phosphotransferase systems modulates synthesis of the KdpFABC potassium transporter in *Escherichia coli*. *J. Mol. Microbiol. Biotechnol.* 25 168–177. 10.1159/000375497 26159077

[B99] Mairet F. (2018). A biomolecular proportional integral controller based on feedback regulations of protein level and activity. *R. Soc. Open Sci.* 5:171966. 10.1098/rsos.171966 29515895 PMC5830784

[B100] Marcus E. A. Sachs G. Wen Y. Scott D. R. (2016). Phosphorylation-dependent and phosphorylation-independent regulation of *Helicobacter pylori* acid acclimation by the Ars RS two-component System. *Helicobacter* 21 69–81. 10.1111/hel.12235 PMC4655181 25997502

[B101] Mascher T. (2014). Bacterial (intramembrane-sensing) histidine kinases: signal transfer rather than stimulus perception. *Trends Microbiol.* 22 559–565. 10.1016/j.tim.2014.05.006 24947190

[B102] Mascher T. Helmann J. D. Unden G. (2006). Stimulus perception in bacterial signal-transducing histidine kinases. *Microbiol. Mol. Biol. Rev.* 70 910–938. 10.1128/MMBR.00020-06 17158704 PMC1698512

[B103] Masilamani R. Cian M. B. Dalebroux Z. D. (2018). *Salmonella* Tol-Pal reduces outer membrane glycerophospholipid levels for envelope homeostasis and survival during bacteremia. *Infect. Immun.* 86:e00173-18. 10.1128/IAI.00173-18 29735519 PMC6013679

[B104] Matson J. S. Livny J. Dirita V. J. (2017). A putative *Vibrio cholerae* two-component system controls a conserved periplasmic protein in response to the antimicrobial peptide polymyxin B. *PLoS One* 12:e0186199. 10.1371/journal.pone.0186199 29020117 PMC5636140

[B105] Mauriello E. M. F. Jones C. Moine A. Armitage J. P. (2018). Cellular targeting and segregation of bacterial chemosensory systems. *FEMS Microbiol. Rev.* 42 462–476. 10.1093/femsre/fuy015 29945173

[B106] May K. L. Lehman K. M. Mitchell A. M. Grabowicz M. (2019). A stress response monitoring lipoprotein trafficking to the outer membrane. *mBio* 10:e00618-19. 10.1128/mBio.00618-19 31138744 PMC6538781

[B107] Mayola A. Irazoki O. Martinez I. A. Petrov D. Menolascina F. Stocker R. (2014). RecA protein plays a role in the chemotactic response and chemoreceptor clustering of *Salmonella enterica*. *PLoS One* 9:e105578. 10.1371/journal.pone.0105578 25147953 PMC4141790

[B108] Minamino T. Kinoshita M. Inoue Y. Morimoto Y. V. Ihara K. Koya S. (2016a). FliH and FliI ensure efficient energy coupling of flagellar type III protein export in *Salmonella*. *Microbiologyopen* 5 424–435. 10.1002/mbo3.340 26916245 PMC4905995

[B109] Minamino T. Morimoto Y. V. Hara N. Aldridge P. D. Namba K. (2016b). The bacterial flagellar type III export gate complex is a dual fuel engine that can use both H+ and Na+ for flagellar protein export. *PLoS Pathog.* 12:e1005495. 10.1371/journal.ppat.1005495 26943926 PMC4778876

[B110] Minamino T. Macnab R. M. (1999). Components of the *Salmonella* flagellar export apparatus and classification of export substrates. *J. Bacteriol.* 181:1388. 10049367 10.1128/jb.181.5.1388-1394.1999 PMC93525

[B111] Mirouze N. Bidnenko E. Noirot P. Auger S. (2015). Genome-wide mapping of TnrA-binding sites provides new insights into the TnrA regulon in *Bacillus subtilis*. *Microbiologyopen* 4 423–435. 10.1002/mbo3.249 25755103 PMC4475385

[B112] Monahan L. G. Harry E. J. (2016). You are what you eat: metabolic control of bacterial division. *Trends Microbiol.* 24 181–189. 10.1016/j.tim.2015.11.007 26690613

[B113] Monzel C. Degreif-Dunnwald P. Gropper C. Griesinger C. Unden G. (2013). The cytoplasmic PASC domain of the sensor kinase DcuS of *Escherichia coli*: role in signal transduction, dimer formation, and DctA interaction. *Microbiologyopen* 2 912–927. 10.1002/mbo3.127 24039243 PMC3892338

[B114] Mukherjee T. Elmas M. Vo L. Alexiades V. Hong T. Alexandre G. (2019). Multiple CheY homologs control swimming reversals and transient pauses in *Azospirillum brasilense*. *Biophys. J.* 116 1527–1537. 10.1016/j.bpj.2019.03.006 30975454 PMC6486476

[B115] Muok A. R. Briegel A. Crane B. R. (2019a). Regulation of the chemotaxis histidine kinase CheA: a structural perspective. *Biochim. Biophys. Acta Biomembr.* 1862:183030. 10.1016/j.bbamem.2019.183030 31374212 PMC7212787

[B116] Muok A. R. Deng Y. Gumerov V. M. Chong J. E. Derosa J. R. Kurniyati K. (2019b). A di-iron protein recruited as an Fe [II] and oxygen sensor for bacterial chemotaxis functions by stabilizing an iron-peroxy species. *Proc. Natl. Acad. Sci. U.S.A.* 116 14955–14960. 10.1073/pnas.1904234116 31270241 PMC6660769

[B117] Muzamal U. Gomez D. Kapadia F. Golemi-Kotra D. (2014). Diversity of two-component systems: insights into the signal transduction mechanism by the *Staphylococcus aureus* two-component system GraSR. *F1000Res* 3:252. 10.12688/f1000research.5512.2 25685323 PMC4314665

[B118] Neira J. L. Hornos F. Cozza C. Camara-Artigas A. Abian O. Velazquez-Campoy A. (2018). The histidine phosphocarrier protein, HPr, binds to the highly thermostable regulator of sigma D protein, Rsd, and its isolated helical fragments. *Arch. Biochem. Biophys.* 639 26–37. 10.1016/j.abb.2017.12.017 29288053

[B119] Nesper J. Hug I. Kato S. Hee C.-S. Habazettl J. M. Manfredi P. (2017). Cyclic di-GMP differentially tunes a bacterial flagellar motor through a novel class of CheY-like regulators. *elife* 6:e28842. 10.7554/eLife.28842 29091032 PMC5677366

[B120] Neumann S. Grosse K. Sourjik V. (2012). Chemotactic signaling via carbohydrate phosphotransferase systems in *Escherichia coli*. *Proc. Natl. Acad. Sci. U.S.A.* 109 12159–12164. 10.1073/pnas.1205307109 22778402 PMC3409764

[B121] Niehus E. Gressmann H. Ye F. Schlapbach R. Dehio M. Dehio C. (2004). Genome-wide analysis of transcriptional hierarchy and feedback regulation in the flagellar system of *Helicobacter pylori*. *Mol. Microbiol.* 52 947–961. 10.1111/j.1365-2958.2004.04006.x 15130117

[B122] Nieto V. Partridge J. D. Severin G. Lai R.-Z. Waters C. Parkinson J. S. (2019). Under elevated c-di-GMP in *E. coli*, YcgR alters flagellar motor bias and speed sequentially, with additional negative control of the flagellar regulon via the adaptor protein RssB. *J. Bacteriol.* 202:e00578-19. 10.1128/JB.00578-19 31611290 PMC6932234

[B123] Nishikino T. Hijikata A. Miyanoiri Y. Onoue Y. Kojima S. Shirai T. (2018). Rotational direction of flagellar motor from the conformation of FliG middle domain in marine *Vibrio*. *Sci. Rep.* 8:17793. 10.1038/s41598-018-35902-6 30542147 PMC6290876

[B124] Osman D. Martini M. A. Foster A. W. Chen J. Scott A. J. P. Morton R. J. (2019). Bacterial sensors define intracellular free energies for correct enzyme metalation. *Nat. Chem. Biol.* 15 241–249. 10.1038/s41589-018-0211-4 30692683 PMC6420079

[B125] Ouyang J. Tian X. L. Versey J. Wishart A. Li Y. H. (2010). The BceABRS four-component system regulates the bacitracin-induced cell envelope stress response in *Streptococcus* mutans. *Antimicrob. Agents Chemother.* 54 3895–3906. 10.1128/AAC.01802-09 20606066 PMC2935011

[B126] Packer H. L. Armitage J. P. (2000a). Behavioral responses of *Rhodobacter sphaeroides* to linear gradients of the nutrients succinate and acetate. *Appl. Environ. Microbiol.* 66 5186–5191. 10.1128/aem.66.12.5186-5191.2000 11097888 PMC92442

[B127] Packer H. L. Armitage J. P. (2000b). Inverted behavioural responses in wild-type *Rhodobacter sphaeroides* to temporal stimuli. *FEMS Microbiol. Lett.* 189 299–304. 10.1111/j.1574-6968.2000.tb09247.x 10930755

[B128] Papenfort K. Bassler B. L. (2016). Quorum sensing signal–response systems in Gram-negative bacteria. *Nat. Rev. Microbiol.* 14 576–588. 10.1038/nrmicro.2016.89 27510864 PMC5056591

[B129] Pappalardo L. Janausch I. G. Vijayan V. Zientz E. Junker J. Peti W. (2003). The NMR structure of the sensory domain of the membranous two-component fumarate sensor (histidine protein kinase) DcuS of *Escherichia coli*. *J. Biol. Chem.* 278 39185–39188. 10.1074/jbc.C300344200 12907689

[B130] Parkinson J. S. Hazelbauer G. L. Falke J. J. (2015). Signaling and sensory adaptation in *Escherichia coli* chemoreceptors: 2015 update. *Trends Microbiol.* 23 257–266. 10.1016/j.tim.2015.03.003 25834953 PMC4417406

[B131] Pflock M. Finsterer N. Joseph B. Mollenkopf H. Meyer T. F. Beier D. (2006). Characterization of the ArsRS regulon of *Helicobacter pylori*, involved in acid adaptation. *J. Bacteriol.* 188 3449–3462. 10.1128/JB.188.10.3449-3462.2006 16672598 PMC1482845

[B132] Porter S. L. Wadhams G. H. Armitage J. P. (2011). Signal processing in complex chemotaxis pathways. *Nat. Rev. Microbiol.* 9 153–165. 10.1038/nrmicro2505 21283116

[B133] Radeck J. Fritz G. Mascher T. (2017). The cell envelope stress response of *Bacillus subtilis*: from static signaling devices to dynamic regulatory network. *Curr. Genet.* 63 79–90. 10.1007/s00294-016-0624-0 27344142

[B134] Randazzo P. Aucouturier A. Delumeau O. Auger S. (2017). Revisiting the in vivo GlnR-binding sites at the genome scale in *Bacillus subtilis*. *BMC Res. Notes* 10:422. 10.1186/s13104-017-2703-9 28835263 PMC5569456

[B135] Rauschmeier M. Schüppel V. Tetsch L. Jung K. (2014). New insights into the interplay between the lysine transporter LysP and the pH sensor CadC in *Escherichia coli*. *J. Mol. Biol.* 426 215–229. 10.1016/j.jmb.2013.09.017 24056175

[B136] Rebbapragada A. Johnson M. S. Harding G. P. Zuccarelli A. J. Fletcher H. M. Zhulin I. B. (1997). The Aer protein and the serine chemoreceptor Tsr independently sense intracellular energy levels and transduce oxygen, redox, and energy signals for *Escherichia coli* behavior. *Proc. Natl. Acad. Sci. U.S.A.* 94 10541–10546. 10.1073/pnas.94.20.10541 9380671 PMC23396

[B137] Ringgaard S. Hubbard T. Mandlik A. Davis B. M. Waldor M. K. (2015). RpoS and quorum sensing control expression and polar localization of *Vibrio cholerae* chemotaxis cluster III proteins *in vitro* and *in vivo*. *Mol. Microbiol.* 97 660–675. 10.1111/mmi.13053 25989366 PMC4646612

[B138] Ringgaard S. Yang W. Alvarado A. Schirner K. Briegel A. (2018). Chemotaxis arrays in *Vibrio* species and their intracellular positioning by the ParC/ParP system. *J. Bacteriol.* 200:e00793-17. 10.1128/JB.00793-17 29531180 PMC6040185

[B139] Rivera-Chavez F. Lopez C. A. Zhang L. F. Garcia-Pastor L. Chavez-Arroyo A. Lokken K. L. (2016). Energy taxis toward host-derived nitrate supports a *Salmonella* pathogenicity Island 1-independent mechanism of invasion. *mBio* 7:e00960-16. 10.1128/mBio.00960-16 27435462 PMC4958259

[B140] Rivera-Chavez F. Winter S. E. Lopez C. A. Xavier M. N. Winter M. G. Nuccio S. P. (2013). *Salmonella* uses energy taxis to benefit from intestinal inflammation. *PLoS Pathog.* 9:e1003267. 10.1371/journal.ppat.1003267 23637594 PMC3630101

[B141] Russell G. Lightman S. (2019). The human stress response. *Nat. Rev. Endocrinol.* 15 525–534. 10.1038/s41574-019-0228-0 31249398

[B142] Rust M. Borchert S. Niehus E. Kuehne S. A. Gripp E. Bajceta A. (2009). The *Helicobacter pylori* anti-sigma factor FlgM is predominantly cytoplasmic and cooperates with the flagellar basal body protein FlhA. *J. Bacteriol.* 191 4824–4834. 10.1128/JB.00018-09 19465658 PMC2715733

[B143] Samanta D. Widom J. Borbat P. P. Freed J. H. Crane B. R. (2016). Bacterial energy sensor Aer modulates the activity of the chemotaxis kinase CheA based on the redox state of the flavin cofactor. *J. Biol. Chem.* 291 25809–25814. 10.1074/jbc.C116.757492 27803157 PMC5207056

[B144] Scheu P. D. Steinmetz P. A. Dempwolff F. Graumann P. L. Unden G. (2014). Polar localization of a tripartite complex of the two-component system DcuS/DcuR and the transporter DctA in *Escherichia coli* depends on the sensor kinase DcuS. *PLoS One* 9:e115534. 10.1371/journal.pone.0115534 25549248 PMC4280142

[B145] Schmitz A. Josenhans C. Suerbaum S. (1997). Cloning and characterization of the *Helicobacter pylori flbA* gene, which codes for a membrane protein involved in coordinated expression of flagellar genes. *J. Bacteriol.* 179 987–997. 10.1128/jb.179.4.987-997.1997 9023175 PMC178789

[B146] Schweinitzer T. Josenhans C. (2010). Bacterial energy taxis: a global strategy? *Arch. Microbiol.* 192 507–520. 10.1007/s00203-010-0575-7 20411245 PMC2886117

[B147] Schweinitzer T. Mizote T. Ishikawa N. Dudnik A. Inatsu S. Schreiber S. (2008). Functional characterization and mutagenesis of the proposed behavioral sensor TlpD of *Helicobacter pylori*. *J. Bacteriol.* 190 3244–3255. 10.1128/JB.01940-07 18245281 PMC2347378

[B148] Shen A. Kamp H. D. Gründling A. Higgins D. E. (2006). A bifunctional O-GlcNAc transferase governs flagellar motility through anti-repression. *Genes Dev.* 20 3283–3295. 10.1101/gad.1492606 17158746 PMC1686605

[B149] Somavanshi R. Ghosh B. Sourjik V. (2016). Sugar influx sensing by the phosphotransferase system of *Escherichia coli*. *PLoS Biol.* 14:e2000074. 10.1371/journal.pbio.2000074 27557415 PMC4996493

[B150] Stannek L. Thiele M. J. Ischebeck T. Gunka K. Hammer E. Volker U. (2015). Evidence for synergistic control of glutamate biosynthesis by glutamate dehydrogenases and glutamate in *Bacillus subtilis*. *Environ. Microbiol.* 17 3379–3390. 10.1111/1462-2920.12813 25711804

[B151] Steinmetz P. A. Wörner S. Unden G. (2014). Differentiation of DctA and DcuS function in the DctA/DcuS sensor complex of *Escherichia coli*: function of DctA as an activity switch and of DcuS as the C4-dicarboxylate sensor. *Mol. Microbiol.* 94 218–229. 10.1111/mmi.12759 25135747

[B152] Strecker A. Schubert C. Zedler S. Steinmetz P. Unden G. (2018). DcuA of aerobically grown *Escherichia coli* serves as a nitrogen shuttle (L-aspartate/fumarate) for nitrogen uptake. *Mol. Microbiol.* 109 801–811. 10.1111/mmi.14074 29995997

[B153] Sule N. Pasupuleti S. Kohli N. Menon R. Dangott L. J. Manson M. D. (2017). The norepinephrine metabolite 3,4-Dihydroxymandelic acid is produced by the commensal microbiota and promotes chemotaxis and virulence gene expression in enterohemorrhagic *Escherichia coli*. *Infect. Immun.* 85:e00431-17. 10.1128/IAI.00431-17 28717028 PMC5607413

[B154] Tang Y. Guest J. R. Artymiuk P. J. Green J. (2005). Switching aconitase B between catalytic and regulatory modes involves iron-dependent dimer formation. *Mol. Microbiol.* 56 1149–1158. 10.1111/j.1365-2958.2005.04610.x 15882410

[B155] Tang Y. Guest J. R. Artymiuk P. J. Read R. C. Green J. (2004). Post-transcriptional regulation of bacterial motility by aconitase proteins. *Mol. Microbiol.* 51 1817–1826. 10.1111/j.1365-2958.2003.03954.x 15009904

[B156] Taylor B. L. Zhulin I. B. Johnson M. S. (1999). Aerotaxis and other energy-sensing behavior in bacteria. *Annu. Rev. Microbiol.* 53 103–128. 10.1146/annurev.micro.53.1.103 10547687

[B157] Terahara N. Inoue Y. Kodera N. Morimoto Y. V. Uchihashi T. Imada K. (2018). Insight into structural remodeling of the FlhA ring responsible for bacterial flagellar type III protein export. *Sci. Adv.* 4:eaao7054. 10.1126/sciadv.aao7054 29707633 PMC5916509

[B158] Tetsch L. Jung K. (2009). The regulatory interplay between membrane-integrated sensors and transport proteins in bacteria. *Mol. Microbiol.* 73 982–991. 10.1111/j.1365-2958.2009.06847.x 19708919

[B159] Tetsch L. Koller C. Haneburger I. Jung K. (2008). The membrane-integrated transcriptional activator CadC of *Escherichia coli* senses lysine indirectly via the interaction with the lysine permease LysP. *Mol. Microbiol.* 67 570–583. 10.1111/j.1365-2958.2007.06070.x 18086202

[B160] Tsang J. Hirano T. Hoover T. R. Mcmurry J. L. (2015). *Helicobacter pylori* FlhA binds the sensor kinase and flagellar gene regulatory protein FlgS with high affinity. *J. Bacteriol.* 197 1886–1892. 10.1128/JB.02610-14 25802298 PMC4420913

[B161] Tsang J. Smith T. G. Pereira L. E. Hoover T. R. (2013). Insertion mutations in *Helicobacter pylori flhA* reveal strain differences in RpoN-dependent gene expression. *Microbiology* 159 58–67. 10.1099/mic.0.059063-0 23154969 PMC3542725

[B162] Tschauner K. Hörnschemeyer P. Müller V. S. Hunke S. (2014). Dynamic interaction between the CpxA sensor kinase and the periplasmic accessory protein CpxP mediates signal recognition in *E. coli*. *PLoS One* 9:e107383. 10.1371/journal.pone.0107383 25207645 PMC4160245

[B163] Unden G. Wörner S. Monzel C. (2016). Cooperation of secondary transporters and sensor kinases in transmembrane signalling: the DctA/DcuS and DcuB/DcuS sensor complexes of *Escherichia coli*. *Adv. Microb. Physiol.* 68 139–167. 10.1016/bs.ampbs.2016.02.003 27134023

[B164] Västermark A. Saier M. H. Jr. (2014). The involvement of transport proteins in transcriptional and metabolic regulation. *Curr. Opin. Microbiol.* 18 8–15. 10.1016/j.mib.2014.01.002 24513656 PMC3999241

[B165] Vuppada R. K. Hansen C. R. Strickland K. A. P. Kelly K. M. Mccleary W. R. (2018). Phosphate signaling through alternate conformations of the PstSCAB phosphate transporter. *BMC Microbiol.* 18:8. 10.1186/s12866-017-1126-z 29351743 PMC5775613

[B166] Wanner B. L. Wilmes-Riesenberg M. R. (1992). Involvement of phosphotransacetylase, acetate kinase, and acetyl phosphate synthesis in control of the phosphate regulon in *Escherichia coli*. *J. Bacteriol.* 174 2124–2130. 10.1128/jb.174.7.2124-2130.1992 1551836 PMC205829

[B167] Ward E. Kim E. A. Panushka J. Botelho T. Meyer T. Kearns D. B. (2019). Organization of the flagellar switch complex of *Bacillus subtilis*. *J. Bacteriol.* 201:e00626-18. 10.1128/JB.00626-18 30455280 PMC6436343

[B168] Whiteley M. Diggle S. P. Greenberg E. P. (2017). Progress in and promise of bacterial quorum sensing research. *Nature* 551 313–320. 10.1038/nature24624 29144467 PMC5870893

[B169] Wiech E. M. Cheng H.-P. Singh S. M. (2015). Molecular modeling and computational analyses suggests that the *Sinorhizobium meliloti* periplasmic regulator protein ExoR adopts a superhelical fold and is controlled by a unique mechanism of proteolysis. *Protein Sci.* 24 319–327. 10.1002/pro.2616 25492513 PMC4353358

[B170] Willett J. W. Crosson S. (2017). Atypical modes of bacterial histidine kinase signaling. *Mol. Microbiol.* 103 197–202. 10.1111/mmi.13525 27618209 PMC5218898

[B171] Winter S. E. Thiennimitr P. Winter M. G. Butler B. P. Huseby D. L. Crawford R. W. (2010). Gut inflammation provides a respiratory electron acceptor for *Salmonella*. *Nature* 467 426–429. 10.1038/nature09415 20864996 PMC2946174

[B172] Witan J. Bauer J. Wittig I. Steinmetz P. A. Erker W. Unden G. (2012). Interaction of the *Escherichia coli* transporter DctA with the sensor kinase DcuS: presence of functional DctA/DcuS sensor units. *Mol. Microbiol.* 85 846–861. 10.1111/j.1365-2958.2012.08143.x 22780562

[B173] Wörner S. Surmann K. Ebert-Jung A. Volker U. Hammer E. Unden G. (2018). Cellular Concentrations of the transporters DctA and DcuB and the sensor DcuS of *Escherichia coli* and the contributions of free and complexed DcuS to transcriptional regulation by DcuR. *J. Bacteriol.* 200:e00612-17. 10.1128/JB.00612-17 29203472 PMC5786699

[B174] Wray L. V. Jr. Zalieckas J. M. Fisher S. H. (2001). *Bacillus subtilis* glutamine synthetase controls gene expression through a protein-protein interaction with transcription factor TnrA. *Cell* 107 427–435. 10.1016/s0092-8674(01)00572-4 11719184

[B175] Wu K. Walukiewicz H. E. Glekas G. D. Ordal G. W. Rao C. V. (2011). Attractant binding induces distinct structural changes to the polar and lateral signaling clusters in *Bacillus subtilis* chemotaxis. *J. Biol. Chem.* 286 2587–2595. 10.1074/jbc.M110.188664 21098025 PMC3024754

[B176] Wuichet K. Zhulin I. B. (2010). Origins and diversification of a complex signal transduction system in prokaryotes. *Sci. Signal.* 3:ra50. 10.1126/scisignal.2000724 20587806 PMC3401578

[B177] Xie Z. Ulrich L. E. Zhulin I. B. Alexandre G. (2010). PAS domain containing chemoreceptor couples dynamic changes in metabolism with chemotaxis. *Proc. Natl. Acad. Sci. U.S.A.* 107 2235–2240. 10.1073/pnas.0910055107 20133866 PMC2836669

[B178] Xiong Y. Yang Z. Zhang J. Li J. Chen P. Xiang Y. (2019). Panning using a phage-displayed random peptide library to identify peptides that antagonize the *Helicobacter pylori* ArsS acid-sensing domain. *Microb. Pathog.* 135:103614. 10.1016/j.micpath.2019.103614 31255726

[B179] Xu D. Jia R. Li Y. Gu T. (2017). Advances in the treatment of problematic industrial biofilms. *World J. Microbiol. Biotechnol.* 33 1–10. 10.1007/s11274-016-2203-4 28409363

[B180] Zarbiv G. Li H. Wolf A. Cecchini G. Caplan S. R. Sourjik V. (2012). Energy complexes are apparently associated with the switch-motor complex of bacterial flagella. *J. Mol. Biol.* 416 192–207. 10.1016/j.jmb.2011.12.027 22210351 PMC4361073

[B181] Zhang Y. Pohlmann E. L. Serate J. Conrad M. C. Roberts G. P. (2010). Mutagenesis and functional characterization of the four domains of GlnD, a bifunctional nitrogen sensor protein. *J. Bacteriol.* 192 2711–2721. 10.1128/JB.01674-09 20363937 PMC2876476

[B182] Zimmer D. P. Soupene E. Lee H. L. Wendisch V. F. Khodursky A. B. Peter B. J. (2000). Nitrogen regulatory protein C-controlled genes of *Escherichia coli*: scavenging as a defense against nitrogen limitation. *Proc. Natl. Acad. Sci. U.S.A.* 97 14674–14679. 10.1073/pnas.97.26.14674 11121068 PMC18977

[B183] Zschiedrich C. P. Keidel V. Szurmant H. (2016). Molecular mechanisms of two-component signal transduction. *J. Mol. Biol.* 428 3752–3775. 10.1016/j.jmb.2016.08.003 27519796 PMC5023499

[B184] Zusman D. R. Scott A. E. Yang Z. Kirby J. R. (2007). Chemosensory pathways, motility and development in *Myxococcus xanthus*. *Nat. Rev. Microbiol.* 5 862–872. 10.1038/nrmicro1770 17922045

